# Proximal recolonization by self-renewing microglia re-establishes microglial homeostasis in the adult mouse brain

**DOI:** 10.1371/journal.pbio.3000134

**Published:** 2019-02-08

**Authors:** Lihong Zhan, Grietje Krabbe, Fei Du, Ian Jones, Meredith C. Reichert, Maria Telpoukhovskaia, Lay Kodama, Chao Wang, Seo-hyun Cho, Faten Sayed, Yaqiao Li, David Le, Yungui Zhou, Yin Shen, Brian West, Li Gan

**Affiliations:** 1 Gladstone Institutes of Neurological Disease, San Francisco, California, United States of America; 2 Department of Neurology, University of California, San Francisco, California, United States of America; 3 Department of Geography, University of Wisconsin-Madison, Madison, Wisconsin, United States of America; 4 Institute for Human Genetics and Department of Neurology, University of California-San Francisco, San Francisco, California, United States of America; 5 Neuroscience Graduate Program, University of California, San Francisco, California, United States of America; 6 Plexxikon Inc., Berkeley, California, United States of America; 7 Helen and Robert Appel Alzheimer’s Disease Institute, Brain and Mind Research Institute, Weill Cornell Medicine, New York, New York, United States of America

## Abstract

Microglia are resident immune cells that play critical roles in maintaining the normal physiology of the central nervous system (CNS). Remarkably, microglia have an intrinsic capacity to repopulate themselves after acute ablation. However, the underlying mechanisms that drive such restoration remain elusive. Here, we characterized microglial repopulation both spatially and temporally following removal via treatment with the colony stimulating factor 1 receptor (CSF1R) inhibitor PLX5622. We show that microglia were replenished via self-renewal, with no contribution from nonmicroglial lineages, including Nestin+ progenitors and the circulating myeloid population. Interestingly, spatial analyses with dual-color labeling revealed that newborn microglia recolonized the parenchyma by forming distinctive clusters that maintained stable territorial boundaries over time, indicating the proximal expansive nature of adult microgliogenesis and the stability of microglia tiling. Temporal transcriptome profiling at different repopulation stages revealed that adult newborn microglia gradually regain steady-state maturity from an immature state that is reminiscent of the neonatal stage and follow a series of maturation programs, including nuclear factor kappa-light-chain-enhancer of activated B cells (NF-κB) activation, interferon immune activation, and apoptosis. Importantly, we show that the restoration of microglial homeostatic density requires NF-κB signaling as well as apoptotic egress of excessive cells. In summary, our study reports key events that take place from microgliogenesis to homeostasis reestablishment.

## Author summary

Microglia are the brain’s primary immune sentinels. After being seeded in the central nervous system (CNS) during embryonic development, microglia form an evenly distributed grid-like population in the adult brain. In the adult, microglia have the remarkable ability to regenerate and restore their population following acute insults, but what drives this homeostatic process is unclear. Here, we addressed this question by studying regeneration following chemical ablation of microglia. In this model, we addressed the origin of repopulating cells, their spatial redistribution characteristics, and the temporal spectrum of gene-expression patterns at different stages of repopulation. We found that the restoration of microglial homeostasis during repopulation is achieved through self-renewal, proximity clonal expansion, and activation of maturation programs.

## Introduction

Microglia are resident parenchymal macrophages in the central nervous system (CNS). Along with the perivascular, meningeal, and choroid plexus macrophages, these cells govern the innate immunity of the CNS [1]. Apart from their primary function in immune surveillance, microglia also perform a multitude of essential CNS tasks: supplying neurotrophic factors, pruning unwanted synapses, and promoting programmed cell death [2]. Consequently, microglia have been implicated in neurodegenerative and neuropsychiatric diseases [2, 3]. In particular, loss of microglial homeostatic control in the form of microgliosis is considered one of the key pathological hallmarks of neurodegenerative diseases.

During embryonic development, myeloid progenitor cells leave the blood island and travel from the yolk sac to colonize the developing fetal brain, which later become microglia [4–6]. In the adult brain, microglia are maintained locally through self-division if the blood brain barrier is kept intact [7]. However, how microglia repopulate the brain following microglial ablation has been intensely debated in the field. It has been shown by multiple groups that the empty microglial niche can be readily repopulated in a matter of days after ablation of more than 90% of cells using colony stimulating factor 1 receptor (CSF1R) inhibitor [8, 9]. In this setting, Elmore and colleagues described a highly proliferative Nestin+ nonmicroglial population, which preceded the appearance of microglial markers, leading to the conclusion that a hidden CNS Nestin+ progenitor pool can rapidly replenish microglia deficiency [8]. Other microglial ablation studies have shown that microglia can be regenerated almost entirely via self-renewal [9, 10]. In addition, Nestin+ progenitors transitioning into adult microglia have not been detected in microglia generated under steady-state conditions [11].

Microglia are territorial sentinels in the CNS. Resting-state microglia evenly tile the parenchyma, forming an intricate cellular grid, with microglial processes constantly surveilling local territories [12]. This tiling pattern is largely determined during seeding of the primitive yolk sac progenitors in the embryo [5]. However, adult microglia have the capacity to migrate over long distances through the optic nerve during microglial repopulation [13], making it challenging to determine whether maintenance of microglial tiling is static or dynamic.

Here, we applied transient 5-Ethynyl-2′-deoxyuridine (EdU) labeling, lineage tracing, as well as bone marrow–transplant experiments during microglial repopulation to resolve the debate over the origin of repopulating cells. Our results indicate that the repopulated microglia are exclusively derived locally from self-renewed microglia rather than a Nestin+ progenitor or circulating myeloid cells. Using a dual labeling system and spatial analyses, we investigated spatial tiling and found that microglial colonies are very stable in the CNS once they are formed.

In addition, to further elucidate the mechanisms underlying microglia homeostasis restoration during repopulation, we performed an RNA-seq–profiling experiment from isolated microglia at various stages of repopulation and showed that the early-stage, adult-born microglia are reminiscent of the neonatal counterpart and undergo a series of transcriptional changes to eventually regain a mature phenotype. In particular, components of immune signaling pathways, particularly nuclear factor kappa-light-chain-enhancer of activated B cells (NF-κB) and interferon, were highly elevated, accompanied by a high rate of apoptosis. Conditional knockout of I-Kappa-B-Kinase Beta (IKKβ), a kinase required for NF-κB activation, impaired microglial repopulation *in vivo*. In summary, our results provide new insights into microglial homeostatic control, demonstrating that the cells have an intrinsic regenerative capacity, activate developmental programs during regeneration, and undergo regulated apoptosis to regain homeostatic density.

## Results

### Release from CSF1 inhibition triggers proliferation of microglia and a nonmicroglial population

**Fig 1:**
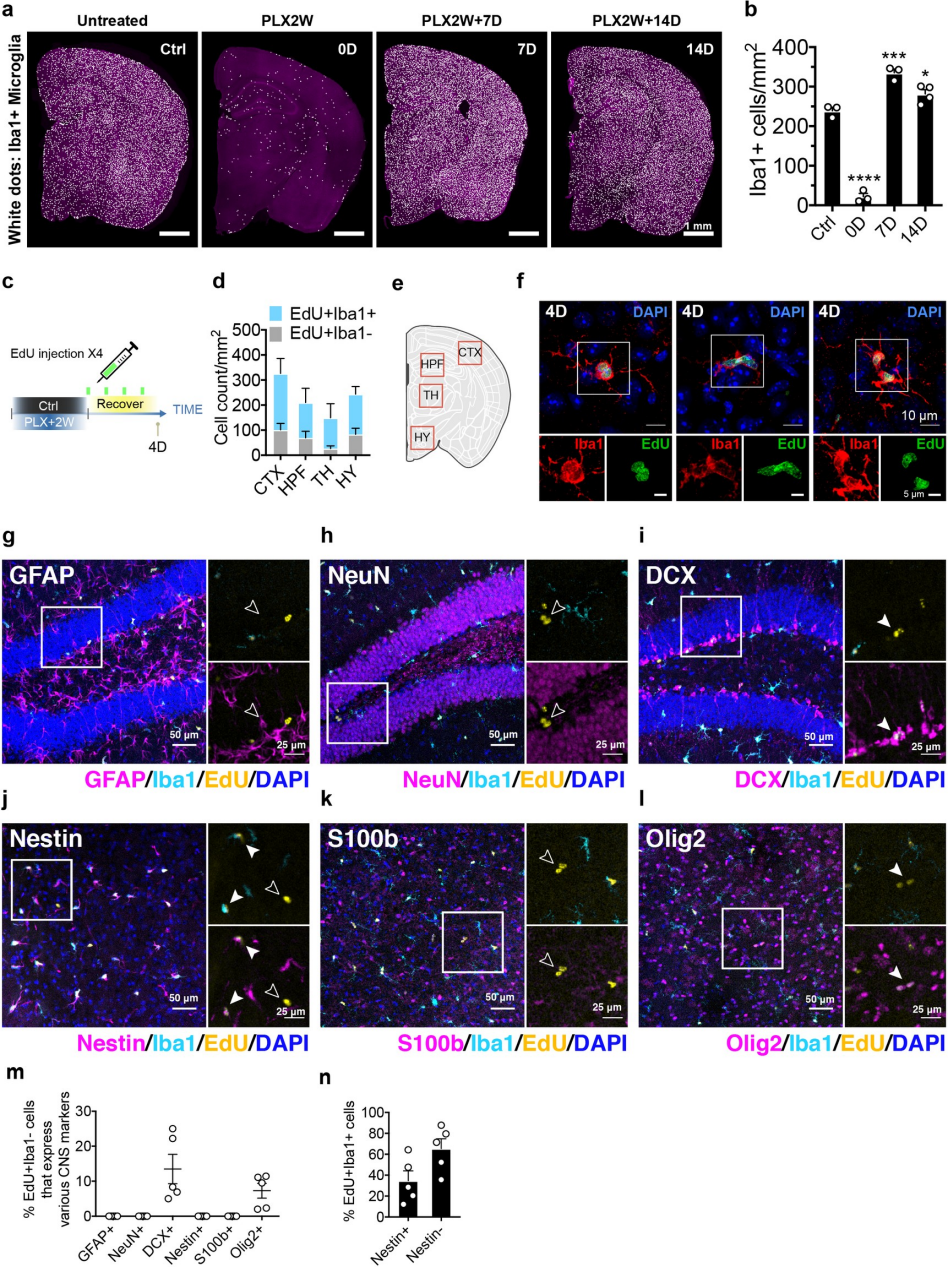
Release from CSF1 inhibition triggers proliferation of microglia and a nonmicroglial population. (a) Stitched image of coronal section showing microglial density at steady state (Ctrl), after 2 weeks of PLX5622 treatment (PLX2W), repopulation for 7 days (PLX2W + 7 D), and repopulation for 14 days (PLX2W + 14 D). C57BL/6J mice were used. Iba1+ cells are shown as microglia. (b) Quantification of microglial density shown in (a). Microglia number was counted and normalized to area of the coronal section (mean ± SEM). Number of C57BL/6J mice (3 Mo) used: Ctrl (*n* = 3), PLX2W (*n* = 3), 7 D (*n* = 3), and 14 D (*n* = 4). One-way ANOVA with Dunnett's multiple comparisons test was used to compare with Ctrl group. (c) Schematic diagram of EdU labeling during microglia repopulation. Microglia were depleted in 3-month-old C57BL/6J mice using PLX5622 diet for 2 weeks. EdU was then administered via IP injection every 24 hours during 4 days of repopulation. (d) Quantification of cell density for EdU+/Iba1+ and EdU+/Iba1− cells (mean ± SEM) from different regions in day 4 repopulating brain. Number of C57BL/6J mice (3 Mo) used: *n* = 5. (e) Diagram showing the sub-brain regions used for quantification (see S2 Fig for representative images). (f) Confocal images showing Iba1+ microglia undergoing mitosis as marked by EdU labeling. (g–l) Confocal microscopy images showing costaining of Iba1, EdU, and a panel with different CNS markers in sections after 4 days of repopulation. EdU+Iba1− cells are highlighted with open arrow heads. EdU+Iba1− cells that are positive for CNS marker are marked by closed arrow heads. GFAP+, NeuN+, and DCX+ cells were imaged from the hippocampal region. Nestin+, S100β+, and Olig2+ cells were imaged from the thalamic region, shown as HPF and TH in panel (e), respectively. (m) Quantification of the percentage of EdU+Iba1− cells expressing different CNS markers out of all EdU+Iba1− cells in mice after 4 days of repopulation (mean ± SEM). Number of animal used: (*n* = 5). (n) Quantification of the percentage of Nestin-positive and Nestin-negative cells out of all EdU+Iba1+ cells. Number of animals used: (*n* = 5). *P* value is summarized as ns (*P* > 0.05), *(*P* ≤ 0.05), **(*P* ≤ 0.01), ***(*P* ≤ 0.001), and ****(*P* ≤ 0.0001). Individual numerical values can be found in S1 Data. CNS, central nervous system; Ctrl, control; CTX, cortex; D, days; DCX, doublecortin; EdU, 5-Ethynyl-2′-deoxyuridine; GFAP, glial fibrillary acidic protein; HY, hypothalamus; HPF, hippocampus; Iba1; IP, intraperitoneal; Mo, months; NeuN, neuronal nuclei; Olig2, oligodendrocyte transcription factor 2; PLX, PLX5622; TH, thalamus.

Microglia are resident immune cells that territorially tile the entire CNS. Microglial homeostasis is tightly controlled under physiological conditions [11]. To investigate the mechanisms underlying microglial homeostatic restoration, we depleted microglia by feeding C56/BL6J mice a diet containing the CSF1R inhibitor PLX5622 (PLX). Administration of PLX for 2 weeks reduced the number of ionized calcium binding adaptor molecule 1 (Iba1+) microglia over 90%. After switching to a regular diet for 7 or 14 days, the microglial population was readily restored and exceeded steady-state level (Fig 1A and 1B). Similar microglial repopulation was observed following diphtheria toxin–mediated selective ablation (S1A Fig). In this model, microglial density was partially restored after 1 day of repopulation and surpassed normal density after 7 days of repopulation (S1B and S1C Fig). These results indicate that repopulation is an inherent property of microglia irrespective of the method of ablation.

To trace the source of repopulating microglia, we injected EdU, a thymidine analog that can be incorporated into newly synthesized DNA, during the first 4 days of microglial repopulation (Fig 1C). Approximately 60% of EdU+ cells were also Iba1+ (Fig 1D and 1E and S2 Fig). Among those cells, we observed pairs of EdU+ microglia that appeared to be undergoing mitosis (Fig 1F), suggesting that microglial repopulation involves self-proliferation. In line with activation of cell cycle re-entry, live imaging of brain slices from microglia reporter mice (CX3CR1^eGFP/+^), which express eGFP under the myeloid promoter CX3CR1, revealed that microglia exhibited increased movement on repopulation day 6 (S3 Fig, S1 Video, and S2 Video). However, roughly 10%–30% EdU+ cells did not express the microglial marker Iba1 (Fig 1D). To identify the EdU+Iba1− cells, we performed staining with Nestin and an array of other common CNS markers (Fig 1G–1L). In contrast to what was previously reported [8], Nestin was not expressed in the EdU+Iba1− cells (Fig 1J, open arrow, and Fig 1M) but was present in roughly 30% EdU+Iba1+ cells (Fig 1J, solid arrow, and Fig 1N), indicating that Nestin may be a transient marker of young microglia. In addition, we also found that a small subset of the EdU+Iba1− cells expressed doublecortin (DCX) or oligodendrocyte transcription factor 2 (Olig2) (Fig 1I, Fig 1L and Fig 1M), markers of neurogenesis and oligodendrocytes, respectively.

### Bone marrow–derived hematopoietic myeloid cells do not contribute to the repopulated microglial pool

**Fig 2:**
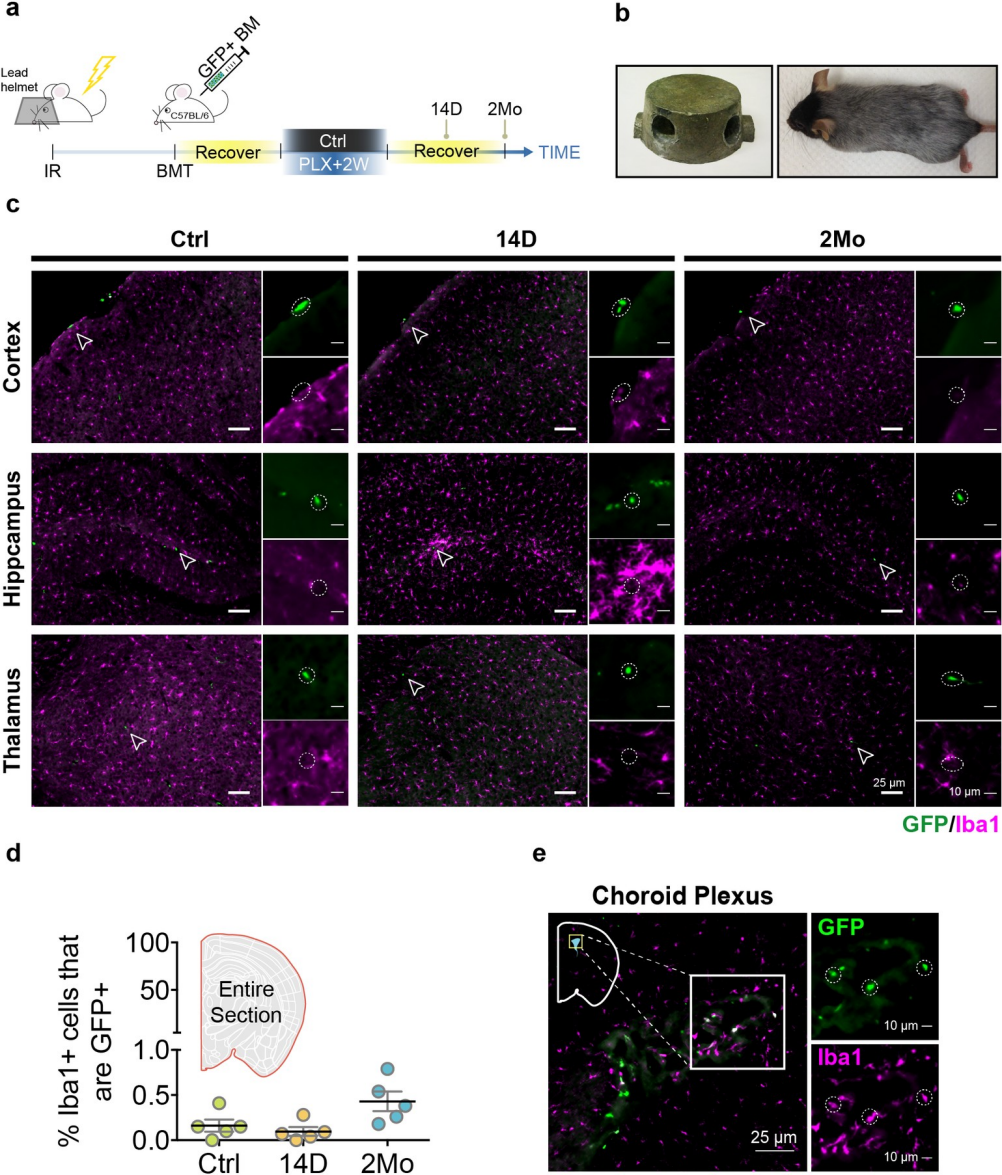
Donor hematopoietic myeloid cells do not contribute to the repopulated microglial pool. (a) Experimental design for the BMT experiment. The BMT mice were treated with 2 weeks of PLX5622 diet and switched to a normal diet for 14 D or 2 Mo for histological analyses. (b) A custom-designed lead helmet used to protect the brain from irradiation damage. Mouse brain was protected by the helmet as shown by the preservation of black fur. (c) Representative images of Iba1+ microglia (red) and GFP+ cells (green) in different brain regions from BMT mice after 14 D and 2 Mo of microglial repopulation. GFP+ cells are highlighted with an open arrowhead and enlarged in the inset highlighted by the dotted circle. (d) Quantification of the percentage of GFP+Iba1+ in repopulated microglia. Quantification was performed using stitched image for the whole coronal section (mean ± SEM). Number of animals used: Ctrl (*n* = 5), 14 D (*n* = 5), and 2 Mo (*n* = 5). (e) GFP+Iba1+ cells shown in choroid plexus and highlighted in the box inset. GFP+Iba1+ cells are highlighted by the dotted circle. Individual numerical values can be found in S1 Data. BMT, bone marrow transplantation; Ctrl, control; D, days; GFP, green fluorescent protein; Iba1, ionized calcium binding adaptor molecule 1; Mo, months; PLX, PLX5622.

To directly test whether circulating monocytes contribute to microglial repopulation after PLX-mediated depletion, we performed bone marrow transplantation (BMT) using wild-type C57BL/6J mice as recipients and ACTB-eGFP mice as donors. The ACTB-eGFP transgenic mice constitutively express eGFP under the control of a chicken beta-actin promoter and cytomegalovirus enhancer [14], enabling the tracing of reconstituted circulating monocytes in the chimeric mice by the presence of eGFP (Fig 2A). Since irradiation of the head can cause artificial monocyte infiltration of the brain [15], mice were fitted with a lead helmet to shield their heads; protection was evidenced by the maintenance of pigmented head hair following irradiation (Fig 2B). Following BMT, mice were subjected to microglial depletion using the PLX diet for 2 weeks and then switched to normal diet for repopulation. On average, chimeric mice showed 70% myeloid reconstitution, as determined by the presence of eGFP in the CD11b+CD45+ population from blood or splenocyte suspension (S4 Fig). However, immunofluorescence staining of coronal brain slices showed very few green fluorescent protein (GFP)+ cells in the parenchyma, either after 14 days or 2 months of repopulation (Fig 2C). Overall, only a small fraction (<0.5%) of the repopulated microglia were GFP+ (Fig 2D). Most of the GFP+Iba1+ myeloid cells were found in the choroid plexus (Fig 2E), which is expected because choroid plexus macrophages are continuously replenished by peripheral monocytes [1]. Consistent with previous studies [9, 10], our findings show that bone marrow–derived hematopoietic myeloid cells are not a major source for the repopulated microglial pool.

### Repopulated microglia are not derived from Nestin+, NG2+, or PDGFra+ progenitors

**Fig 3:**
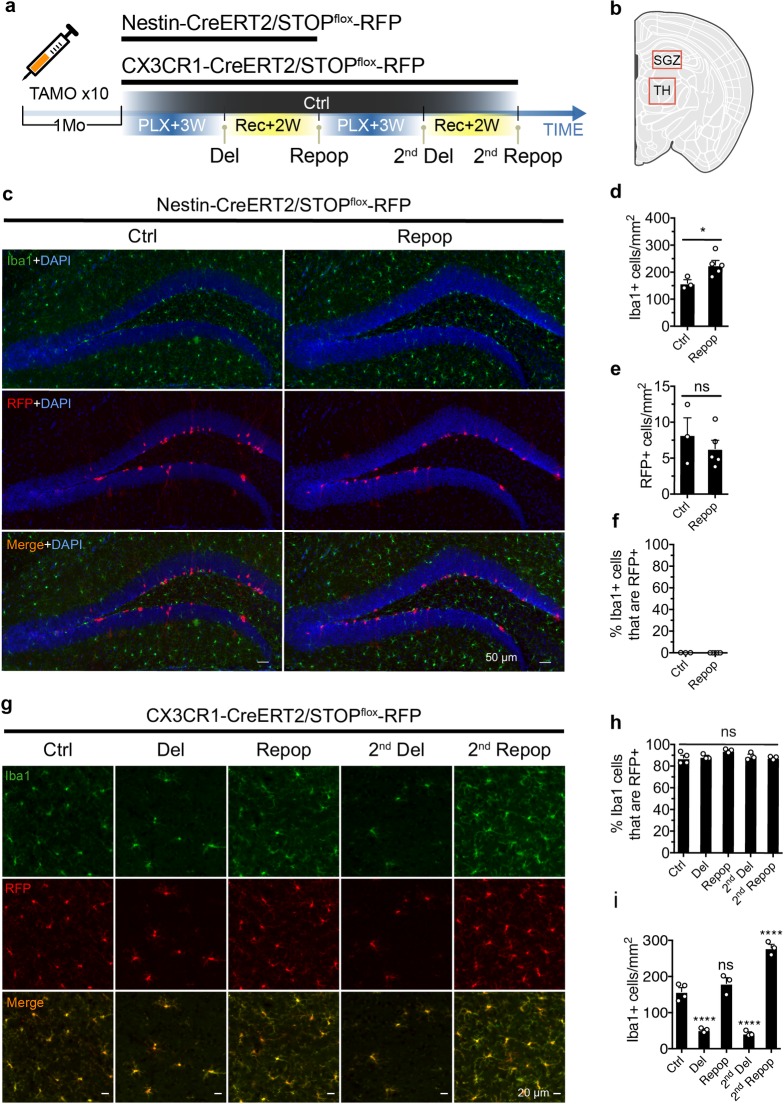
Repopulated microglia are exclusively derived from CX3CR1+ cell lineage. (a) Schematic diagram of lineage tracing experiment for repopulated microglia. Nestin-CreERT2/STOP^flox^-RFP mice (4 Mo) or CX3CR1-CreERT2/STOP^flox^-RFP mice (7–9 Mo) were given 2 mg tamoxifen daily via IP injection for 10 days. Two weeks after the last tamoxifen administration, mice were subjected to 3 weeks of PLX treatment (Del) before switching back to normal diet for 2 weeks (Repop). This microglial depletion/repopulation regimen was repeated for a second round (2nd Del, 2nd Repop) for the CX3CR1-CreERT2/STOP^flox^-RFP mice. (b) Diagram showing the SGZ region and TH region used for imaging and quantification performed in the Nestin-CreERT2/STOP^flox^-RFP mice (c–f) and CX3CR1-CreERT2/STOP^flox^-RFP mice (g, i), respectively. (c) Representative images showing Nestin+ lineage (RFP+, red) and microglia (Iba1+, green) at the SGZ before and after microglial repopulation. Nestin-CreERT2/STOP^flox^-RFP mice (4 Mo) were treated as described in panel (a), with a single round of microglial depletion and repopulation. (d) Quantification of Iba1+ microglial cell density before and after repopulation (mean ± SEM). Number of animals used: Ctrl (*n* = 3) and Repop (*n* = 4). (e) Quantification of cell density of Nestin lineage (RFP+) before and after microglial repopulation (mean ± SEM). (f) Quantification of Iba1+ microglia that express Nestin before and after microglial repopulation (mean ± SEM). Unpaired *t* test was used to compute statistical differences (d, e). (g) Representative images showing Iba1+ (green) and RFP (red) after depletion and repopulation. Images were taken from the TH region. (h) Quantification of the percentage of Iba1+ microglia that express RFP (mean ± SEM). Numbers of animals used: Ctrl (*n* = 4), 1st Del (*n* = 3), 1st Repop (*n* = 3), 2nd Del (*n* = 3), and 2nd Repop (*n* = 3). One-way ANOVA was used to assess statistical differences among the groups. (i) Quantification of Iba1+ microglial density after different treatments (mean ± SEM). One-way ANOVA with Dunnett's multiple comparisons test was used by comparing to the Ctrl group. *P* value is summarized as ns (*P* > 0.05), *(*P* ≤ 0.05), **(*P* ≤ 0.01), ***(*P* ≤ 0.001), and ****(*P* ≤ 0.0001). Individual numerical values can be found in S1 Data. Ctrl, control; CreERT2, tamoxifen-inducible Cre recombinase; CX3CR1, CX3C chemokine receptor 1; Del, deletion; Iba1, ionized calcium binding adaptor molecule 1; IP, intraperitoneal; Mo, months; ns; PLX, PLX5622; Repop, repopulation; RFP, red fluorescent protein; SGZ, subgranular zone; TH, thalamus.

We and others have observed that Nestin, a marker of neuroprogenitor cells, is transiently expressed in early-stage newborn microglia [8, 10]. To directly test if Nestin+ progenitors can also contribute to the repopulated microglial pool, as proposed previously [8], we performed fate-mapping experiments using Nestin-CreERT2 [16] and the STOP^flox^-RFP reporter to label Nestin+ progenitor cells with red fluorescent protein (RFP) (Fig 3A). Upon tamoxifen treatment, removal of the STOP cassette by the CreERT2 recombinase triggers RFP expression under a constitutive CAG promoter, thus ensuring any descendent cell divided from the labeled microglia will also be RFP+. Neuronal precursor cells were labeled with RFP in the subgranular zone (SGZ), as expected (Fig 3B and 3C). After 3 weeks of PLX treatment, followed by 2 weeks of microglial repopulation (Fig 3D), the number of RFP+ cells remained unchanged (Fig 3E), and none of the repopulated microglia expressed RFP (Fig 3F). Thus, Nestin+ progenitors do not contribute to the repopulated microglia pool. Given that a small proportion of EdU+Iba1− express Olig2+ during microglia repopulation (Fig 1L), we also tested whether oligodendrocyte precursor cells (OPCs) could give rise to microglia. STOP^flox^-RFP reporter mice were crossed with mice that express tamoxifen-inducible Cre recombinase under either the platelet derived growth factor receptor alpha (PDGFra) promoter [17] or the neural/glial antigen 2 (NG2) promoter [18]. While PLX treatment did not affect the number of PDGFra+ progenitor cells labeled with RFP, none of the RFP+ cells became repopulated microglia (S5A–S5D Fig). Similar results were seen in the NG2-CreERT2 mice (S5E–S5H Fig). Taken together, these lineage-tracing experiments indicate that the origin of adult microgliogenesis is distinct from that of neurogenesis or oligodendrogenesis.

### Repopulated microglia are derived from a CX3CR1+ cell lineage

To further investigate the origin of the repopulated microglia, we performed fate-mapping analysis using a combination of the STOP^flox^-RFP reporter [19] and the tamoxifen inducible myeloid-specific driver CX3CR1-CreERT2 [20] to genetically label adult microglia (Fig 3A). Treating mice for 10 days with 2 mg tamoxifen/day resulted in approximately 85% efficient microglial labeling (Fig 3G and 3H), similar to other studies [20, 21]. If microglia are repopulated from a nonmicroglial source, this labeling rate would be expected to drop following ablation. To test this, we subjected labeled mice to 2 rounds of microglial depletion and repopulation. We found that the percentage of RFP expression in repopulated microglia remained constant at approximately 85% for both repopulation rounds (Fig 3G and 3H). Thus, the repopulated microglia are almost exclusively derived from the remaining CX3CR1+ microglia, most likely via self-renewal. Notably, the second round of depletion did not exhaust the regenerative capacity of microglia, further supporting the self-renewal property of this population (Fig 3I). Results from the CX3CR1+ lineage tracing suggests that microglia self-renewal alone is sufficient to repopulate the entire parenchyma after acute ablation, as also found by Bruttger and colleagues [10] as well as Huang and colleagues [9].

### Repopulated microglia are organized in distinct cell clusters

**Fig 4:**
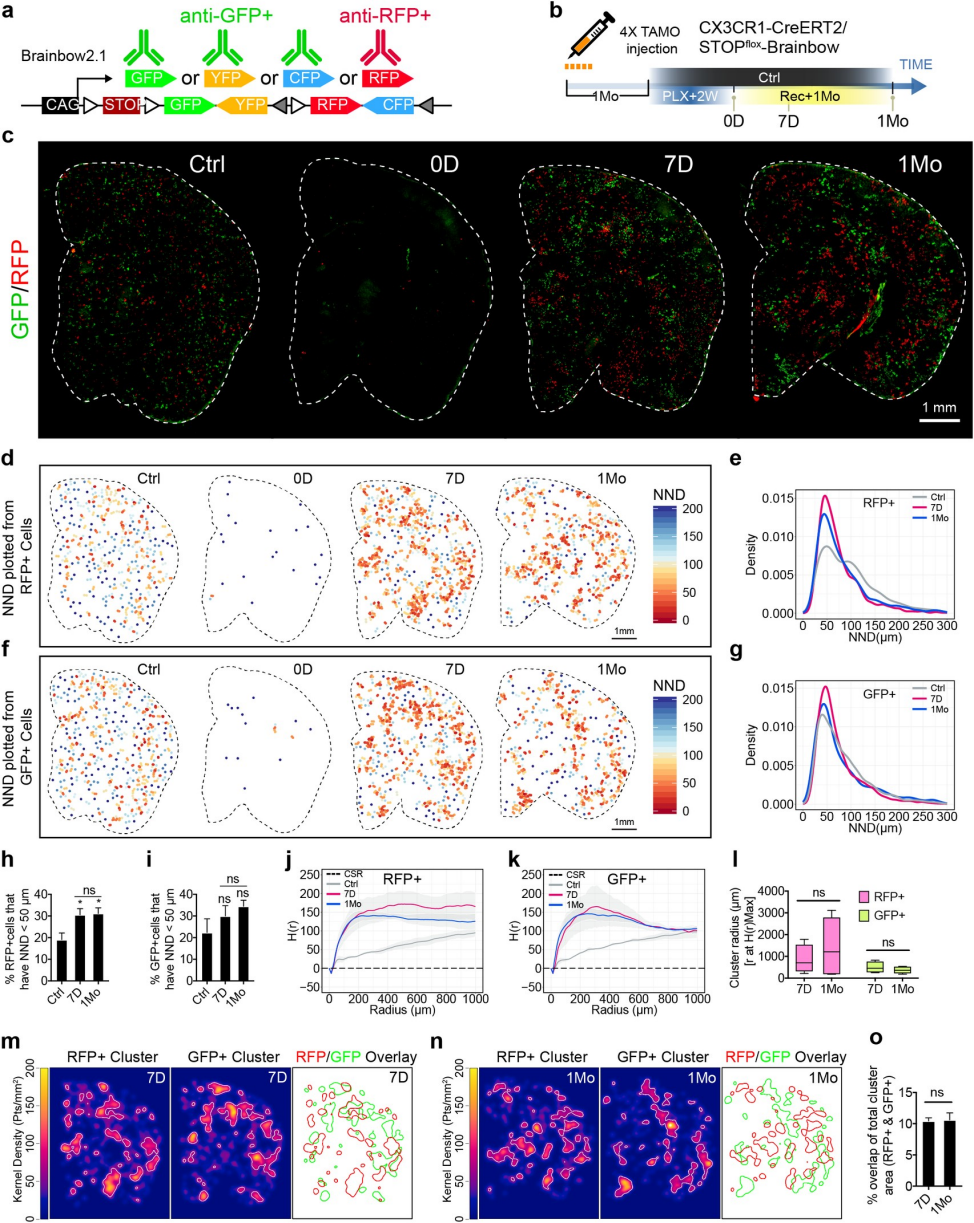
Repopulated microglia form stable clusters with minimal migratory diffusion. (a) Immunofluorescent staining strategy to visualize RFP+ or GFP+ microglia using the Brainbow reporter. (b) Experimental scheme to sparsely label microglia with Brainbow reporter. The CX3CR1-CreERT2/STOP^flox^-Brainbow mice (7–8 Mo) were given a daily dose of 2 mg tamoxifen per animal via IP injection for 4 days. Labeled mice were subjected to 2 weeks of PLX treatment before switching to normal diet for 7 D or 1 Mo. (c) Representative images from coronal sections of RFP+ cells (red) and GFP+ cells (green). Parenchyma outline is visualized with white dotted line. (d) Spatial heatmap of NND reconstructed from RFP+ cells. Each dot represents a single cell color-coded by NND score. (e) Density plot showing NND distribution from RFP+ cells. (f) Spatial heatmap of NND reconstructed from GFP+ cells. (g) Density plot showing NND distribution from GFP+ cells. (h) Quantification of the percentage of RFP+ cells that have equal or less than 50 μm NND (mean ± SEM). Animals used: Ctrl (*n* = 5), 7 D (*n* = 4), and 1 Mo (*n* = 5). One-way ANOVA with Dunnett's multiple comparisons test was used by comparing to the Ctrl group. Sidak's multiple comparisons test was used to compare 7 D and 1 Mo. (i) Quantification of the percentage of GFP+ cells that have equal or less than 50 μm NND. Statistical analysis was the same as (h). (j, k) Plot of Ripley’s H-function analysis on RFP+ (h) and GFP+ (i) cell-clustering patterns. Black dotted line represents CSR, i.e., absence of clustering pattern. Average H(r) value from each group were plotted. Grey ribbon shades represent SEM. Animals used: Ctrl (*n* = 5), 7 D (*n* = 4), and 1 Mo (*n* = 5). (l) Cluster domain size estimation from H(r)Max. Box-whisker plot of cluster domain size estimation from H(r)Max (whisker: max and min; box: 25 and 75 percentile). Unpaired *t* test was used. (m, n) 2D kernel density map showing cluster interaction between RFP+ and GFP+ cells. Representative sample from 7 D (m) and 1 Mo (n) were plotted. White line marks the border of isolated cluster domains based on the top 10% of the highest kernel density. Overlay of the isolated RFP+ and GFP+ cluster contours are delineated with red and green lines, respectively. (o) Quantification of the percentage of overlapping area of RFP+ and GFP+ clusters with respect to total RFP+ and GFP+ cluster area (mean ± SEM). Animals used: 7 D (*n* = 4); 1 Mo (*n* = 5). Unpaired *t* test was used. *P* value is summarized as ns (*P* > 0.05), *(*P* ≤ 0.05), **(*P* ≤ 0.01), ***(*P* ≤ 0.001), and ****(*P* ≤ 0.0001). Individual numerical values can be found in S1 Data. CreERT2, tamoxifen-inducible Cre recombinase; CSR, complete spatial randomness; Ctrl, control; CX3CR1, CX3C chemokine receptor 1; D, days; GFP, green fluorescent protein; IP, intraperitoneal; Mo, months; NND, nearest neighbor distance; PLX, PLX5622; RFP, red fluorescent protein.

In order to investigate the tiling behavior of microglia during repopulation, we applied Microfetti labeling [22] using the Brainbow2.1 reporter [23] and CX3CR1-CreERT2. Microfetti utilizes stochastic labeling of microglia with 4 fluorescent proteins: GFP, yellow fluorescent protein (YFP), cyan fluorescent protein (CFP), and RFP. Due to fixative-induced inactivation of native fluorescent signals, we used an anti-GFP antibody to detect GFP/CFP/YFP and an anti-RFP antibody to detect RFP (Fig 4A). After 2 weeks of PLX treatment, the CX3CR1-CreERT2/STOP^flox^-Brainbow mice were switched to a normal diet for repopulation over an extended period (up to 1 month) to examine the long-term clonal behavior of newborn microglia (Fig 4B). At a low dose of tamoxifen, uniform and sparse labeling of microglia with the Brainbow reporter was observed via immunostaining of GFP and RFP (Fig 4C). On average, 6.9% microglia were labeled with RFP, while 9.9% were labeled with GFP. PLX-mediated depletion removed over 90% of Iba1+ microglia (S6A and S6B Fig) and retained a few remaining RFP+ or GFP+ cells at day 0 (Fig 4C). After 7 days of repopulation, clonal clusters of both RFP+ and GFP+ microglia were observed (Fig 4C), indicating that adult microgliogenesis occurs via clonal expansion.

### Repopulated microglia form stable clusters with minimal migratory diffusion

To study the properties of the clusters and their dynamics over time, we assigned nearest neighbor distance (NND) values to individual cells based on their distance away from the nearest neighbor. In this analysis, cells inside a tightly formed cluster will have low NND values, whereas dispersed cells will have high NND values. The NND-encoded heatmap offers an overview of the clustering patterns and shows that labeled cells form patches of clusters after repopulation, as expected from self-renewal (Fig 4D and 4F). The density of NNDs near the 50-μm interval increased during repopulation, indicative of colony formation (Fig 4E and 4G). Because unlabeled Iba1+ microglia in naive mice have an NND value of approximately 50 μm (S6C Fig), we used NND of less than 50 μm to quantify changes in clustering patterns compared to controls. Using this criterion, there was an increase in the percentage of RFP+ cells with NND values less than 50 μm at 7 days and 1 month following ablation compared to the Ctrl group (Fig 4H). Interestingly, there was no difference in clustering pattern between 7 days and 1 month, suggesting that once the clusters are formed, they remain stable. Similar results were obtained by analyzing GFP+ cells (Fig 4F and 4G), although NND analysis did not reach statistical significance (Fig 4I), most likely because 3 fluorophores (CFP/YFP/GFP) were combined in this group, resulting in denser labeling in the controls.

To further confirm the results obtained from the NND analysis, we next used Ripley’s K-function analysis and its derivative H-function to survey the spatial points in a defined field with varying radii. Consistent with our NND analysis, the H-function analysis showed that the 7 day and 1 month groups shared similar clustering patterns that were quite different from the Ctrl group for both RFP+ and GFP+ cell populations (Fig 4J and 4K). Furthermore, quantification of the radius of H-function at the maximum score [24] showed that the cluster sizes were indistinguishable between the 7 day and 1 month groups (Fig 4L), suggesting that the size of both RFP+ and GFP+ clusters remain relatively constant over time.

We next investigated whether distinctive clusters could merge with their neighbor through cell migration and spread. Using 2D kernel density maps generated by applying kernel smoothing to actual microglia spatial points, we examined the cluster interaction between RFP+ and GFP+ cells. In order to distinguish cluster boundaries, we used top 10% density from the kernel density map as a threshold. This method allows us to artificially outline cluster boundaries for both RFP+ and GFP+ cells at 7 days (Fig 4M) and 1 month of repopulation (Fig 4N). We then measured the relative overlapping area between RFP+ and GFP+ clusters: if a cluster merged into neighboring clusters, the overlapping area would increase accordingly. The analysis showed a consistent level of overlapping area between the 7 day and 1 month groups (Fig 4O), suggesting that the clusters’ territories are stable with minimal migratory diffusion during the repopulation process. Together, these data indicate adult newborn microglia redistribute spatially, form distinct clusters, and maintain stable tiling pattern over long periods of time.

### Regenerating microglia recapitulate immature signatures of neonatal development

**Fig 5:**
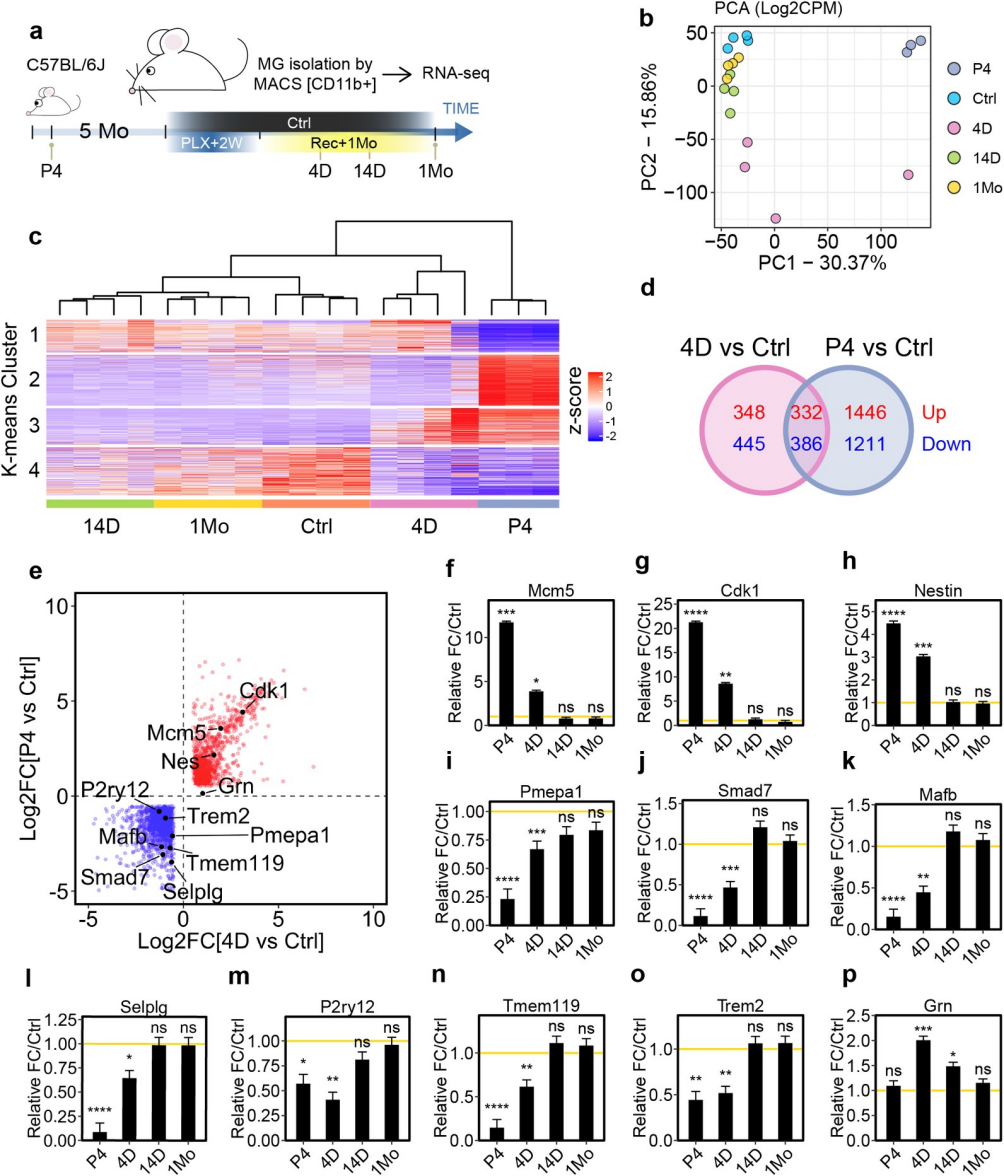
Adult newborn microglia progressively restore homeostatic maturity from a unique immature state. (a) Schematic design for the RNA-seq experiment. C56BL/6 mice (5 Mo) were treated with 2 weeks of PLX5622 diet and switched to a normal diet for 4 days (4 D), 14 days (14 D), or 1 month (1 Mo). (b) PCA analysis of relative gene expression variance from control (Ctrl), P4 neonatal microglia (P4), and after 4 days (4 D), 14 days (14 D), and 1 month (1 Mo) of repopulation. The largest principle components, PC1 and PC2, were used to plot the data. (c) Heatmap showing k-means clustering (k = 4) for relative gene expression. Dendrogram indicates the hierarchical clustering of each biological replicates. Number of animals used: Ctrl (*n* = 4), 4 D (*n* = 4), 14 D (*n* = 4), 1 Mo (*n* = 4), and P4 (*n* = 3). (d) Venn diagram comparing differentially expressed genes between 4 D adult newborn microglia and P4 neonatal microglia. DE genes: Log2FC ≥ 1 or ≤ −1 and FDR < 0.05 in comparison to unperturbed adult microglia (Ctrl). Up-regulated genes and down-regulated genes are shown in red and blue, respectively. (e) Scatter plot showing DE genes shared by 4 D and P4 microglia. DE genes were calculated in comparison to Ctrl microglia with Log2FC ratio greater or less than 1 with FDR < 0.05. DE genes that are up-regulated in both 4D and P4 microglia are shown as red dots, while down-regulated genes are shown as blue dots. (f–p) Relative gene expression of *Mcm5* (f), *Cdk1* (g), *Nestin* (h), *Pmepa1*(i), *Smad7* (j), *Mafb* (k), *Selplg* (l), *P2ry12* (m), *Tmem119* (n), *Trem2* (o), and *Grn* (p). Relative fold change was calculated in comparison to untreated control microglia. The yellow lines (y = 1) indicate normalized baseline expression of control. FDR is summarized as ns (*P* > 0.05), *(*P* ≤ 0.05), **(*P* ≤ 0.01), ***(*P* ≤ 0.001), and ****(*P* ≤ 0.0001). Individual numerical values can be found in S1 Data. Ctrl, control; D, days; DE, differentially expressed; FDR, false discovery rate; Log2FC, log2 transformed fold change; Mo, months; PCA, principal component analysis; PLX, PLX5622.

We next performed RNA-seq to profile adult newborn microglial transcriptome at different stages of repopulation after PLX treatment (S1A Table and S2 Data). After 2 weeks of PLX treatment, repopulated microglia were collected at 4 days (4 D), 14 days (14 D), and 1 month (1 Mo) and compared to immature microglia isolated from postnatal day 4 (P4) pups (Fig 5A) [25, 26]. Microglia were isolated by the magnetic-activated cell sorting (MACS) method using magnetic beads conjugated with CD11b antibody and showed no significant presence of other major CNS cell types based on their specific marker expression (S7A Fig). We confirmed the RNA-seq data via quantitative PCR (qPCR) based on a few differentially expressed genes (S7B–S7D Fig). Dimensional reduction with principal component analysis (PCA) showed biological replicates from each treatment group were very closely related, as indicated by the clustering (Fig 5B). There was a clear separation of the adult microglia (Ctrl) and immature microglia (P4) clusters as expected (Fig 5B). Interestingly, early-stage newborn microglia (4 D) samples clustered between the Ctrl and P4 microglia clusters, while medium- (14 D) and late-stage (1 Mo) microglia clustered closer to control adult microglia (Ctrl), suggesting that newborn microglia undergo progressive restoration toward a steady-state microglial transcriptional profile.

Next, we applied k-means clustering to arbitrarily categorize all genes into 4 distinct clusters. Among all the repopulating microglia groups, the earliest stage (4 D) showed the most transcriptional difference from Ctrl (Fig 5C). Interestingly, genes in cluster 3 and 4 shared expression directionality between 4 D repopulated microglia and P4 neonatal microglia (Fig 5C). This suggests that the newly regenerated microglia might have partially reverted back to an immature developmental state. To assess the similarity of microglia isolated from different repopulation stages, we employed a Poisson distance matrix based on gene expression profiles. Interestingly, as repopulation time goes by, newborn microglia (4 D, 14 D, and 1Mo) showed decreasing similarity with P4 neonatal microglia but increasing similarity with steady-state microglia (Ctrl) (S8A Fig), suggesting that newborn microglia gradually regains maturity. In particular, we found 4 D microglia shared 34% similarity with P4 neonatal microglia and 55% similarity with Ctrl microglia (S8A Fig). This suggests that 4 D microglia adopt a unique immature transcriptome signature that partially overlaps with that of neonatal microglia.

We next performed differentially expressed (DE) gene analysis. Roughly half of the DE genes found in 4 D microglia were also present in P4 microglia (Fig 5D and 5E). Among all of the up-regulated genes shared between 4 D and P4 microglia, many are involved in cell cycle regulation (S8B and S8C Fig), including *Mcm5* and *Cdk1* (Fig 5F and 5G). Importantly, *Nestin* was also elevated at this early stage (Fig 5H) but returned to control levels after 14 days, further validating our earlier observation that Nestin was an immature marker expressed in newborn microglia rather than a bona fide microglial progenitor marker. On the other hand, among all of the down-regulated genes shared between 4 D and P4 microglia, a large fraction is involved in Mitogen Activated Protein Kinase (MAPK) pathway and transforming growth factor β (TGF-β) signaling (S8D and S8E Fig). TGF-β signaling has been shown to be critically important for microglial development and homeostatic maintenance [26], and TGF-β signaling-related genes such as *Pmepa1* and *Smad7* were found to be down-regulated in 4 D microglia (Fig 5I and 5J).

### Newborn microglia re-express mature markers at later stages of repopulation

In addition, previously established mature microglia genes such as *MafB* and *Selplg* [25] and *P2ry12* and *Tmem119* [25–27] were also down-regulated in 4 D microglia but restored after 14 days (Fig 5K–5N). These results indicate that newborn microglia regain maturity over time, and this was validated in morphological analysis and immunostaining of P2ry12 and Tmem119 protein expression in separate experiments (S9 Fig). Interestingly, disease-associated microglial genes such as *Trem2* [28] and *Grn* [29] were also differentially expressed in 4 D microglia (Fig 5O and 5P). Collectively, the results gathered from the RNA-seq analyses suggest that adult microgliogenesis involves maturation steps that partially recapitulate development.

### Adult newborn microglia exhibit distinctive temporal transcriptome profiles

**Fig 6:**
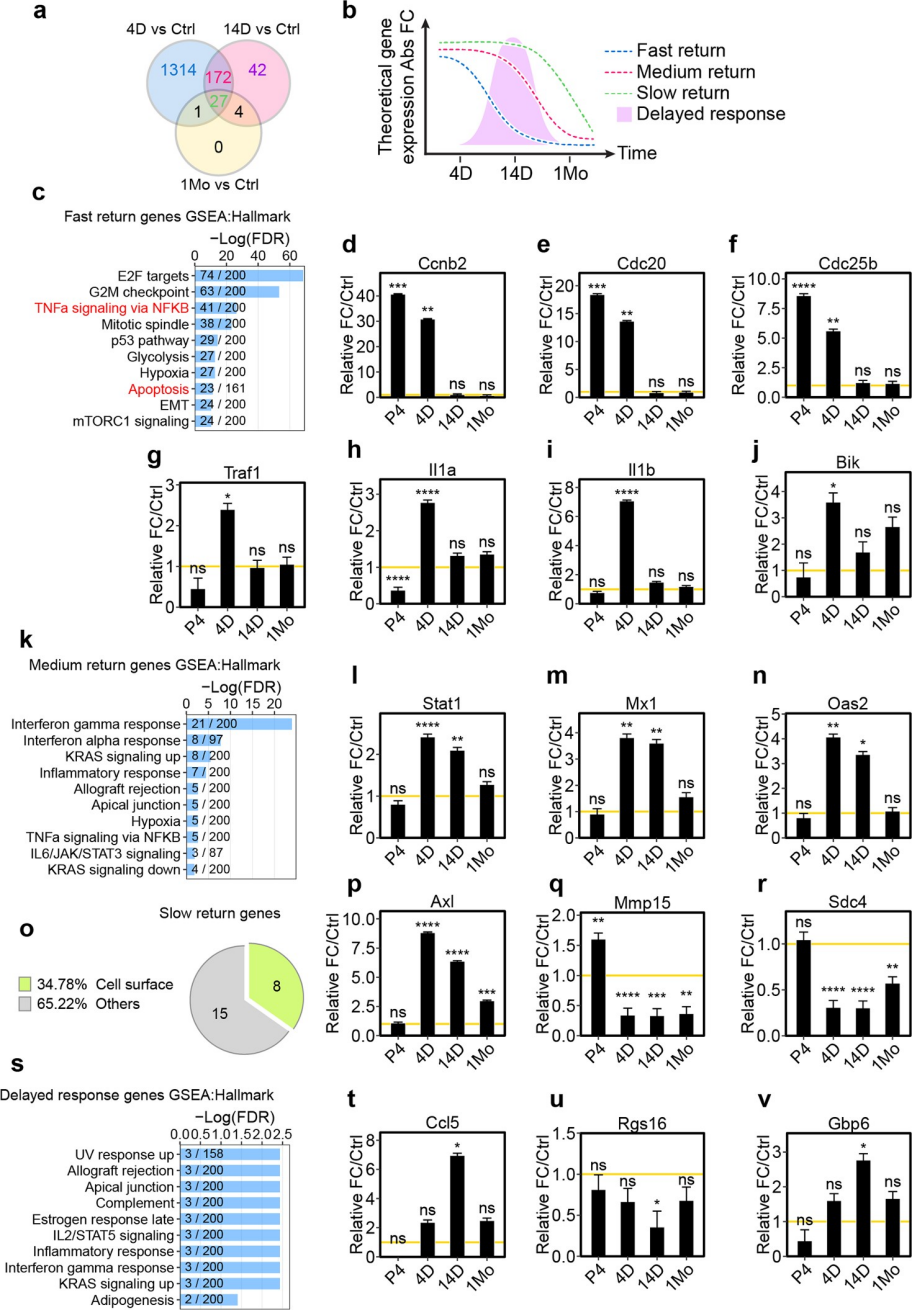
Adult newborn microglia engage a stepwise program to restore their steady-state gene signature. (a) Venn diagram comparing DE genes among 4 D, 14 D, and 1 Mo adult newborn microglia. DE genes were calculated in comparison to Ctrl microglia with Log2FC ratio greater or less than 1 with FDR < 0.05. (b) Schematic diagram illustrating gene sets with differential rates of homeostatic return. (c) GSEA analysis of fast-return genes using the hallmark gene set. The top 10 most-enriched pathways are shown. The number of genes identified in the RNA-seq is shown as numerator. The number of total genes curated for the specific term is shown as denominator. (d–j) Relative gene expression of *Cdcnb2* (d), *Cdc20* (e), *Cdc25b* (f), *Traf1* (g), *Il1a* (h), *Il1b* (i), and *Bik* (j). (k) GSEA analysis on medium-return genes using the hallmark gene set. (l–n) Relative gene expression of *Stat1* (l), *Mx1* (m), and *Oas2* (n). (o) GO (cellular component) analysis on slow-return genes. (p–r) Relative gene expression of *Axl* (p), *Mmp15* (q), and *Sdc4* (r). (s) GSEA analysis on “delayed response” genes using the hallmark gene set database. (t–v) Relative gene expression of *Ccl5* (t), *Rgs16* (u), and *Gbp6* (v). For all gene expression graphs, relative fold change was calculated in comparison to untreated Ctrl microglia. Yellow lines (y = 1) indicate normalized baseline expression of control. FDR is summarized as ns (*P* > 0.05), *(*P* ≤ 0.05), **(*P* ≤ 0.01), ***(*P* ≤ 0.001), and ****(*P* ≤ 0.0001). Individual numerical values can be found in S1 Data. Ctrl, control; DE, differentially expressed; FDR, false discovery rate; GO, gene ontology; GSEA, gene set enrichment analysis; Log2FC, log2 transformed fold-change.

To further dissect the underlying programs associated with microglial maturation, we classified 4 different gene sets based on their distinctive differential expression patterns during each of the repopulation stages (Fig 6A and 6B; log2 ratio of ±1, false discovery rate [FDR] < 0.05). Fast-returning genes were differentially expressed only in 4 D microglia but returned to control levels in 14 D microglia (S1B Table). Medium-returning genes were differentially expressed in 4 D and 14 D microglia but not in 1 Mo microglia (S1C Table). Slow-returning genes were differentially expressed in all repopulation stages (S1D Table). In addition, we also uncovered a fourth set of genes that were only differentially expressed in 14 D microglia, which we refer to as delayed-response genes (S1E Table). We applied gene set enrichment analysis (GSEA) with molecular signatures database (MSigDB) on these 4 gene sets [30, 31]. Comparison of the fast-returning genes with the hallmark gene data set in MSigDB revealed enrichment of genes involved in cell division such as E2F target genes and genes involved in the G2M checkpoint or mitotic spindle (Fig 6C). Indeed, cell cycle–related genes such as *Ccnb2*, *Cdc20*, and *Cdc25b* were highly expressed in 4 D microglia but returned to homeostatic levels in 14 D microglia (Fig 6D–6F). This result further supports our earlier observation that re-entry to the cell cycle is an early step in microglial repopulation (Fig 1F).

### Adult newborn microglia engage a stepwise program to restore homeostatic gene expression

Gene enrichment analyses of fast-returning genes revealed a high degree of overlap with NF-κB pathway-related genes (41 out of 200 genes) such as *Traf1*, *Il1a*, and *Il1b* (Fig 6G–6I). Both *Il1a* and *Il1b* are also associated with cell death. Interestingly, apoptosis-related genes (23 out of 161 genes) were also among the most enriched genes, including *Bik* (Fig 6J). Next, we examined the medium-return genes. Gene enrichment analysis showed strong representation of genes involved in interferon (IFN) pathways such as *Stat1*, *Mx1*, and *Oas2* (Fig 6K–6N). The enrichment of IFN genes indicates inflammatory activation during early and middle phases of microglial repopulation that was resolved after 1 month. The gene enrichment analysis for the slow-return gene set did not have statistically significant overlapping pathways. However, gene ontology analysis focused on cellular components revealed 34% of slow-return genes are cell surface related (Fig 6O). Among the most up-regulated genes was *Axl* (Fig 6P), an important receptor involved in microglial phagocytic clearance of dead cells [32]. Other down-regulated genes include *Mmp15* (Fig 6Q), a metalloproteinase involved in extracellular matrix remodeling [33], and *Sdc4* (Fig 6R), which is an important cell surface adhesion molecule involved in cell activation and migration [34].

The delayed-response gene set revealed modest enrichment of GSEA hallmark genes (Fig 6S). Interestingly, immune-related genes across different inflammatory functions were highly over-represented. *Ccl5*, also known as RANTES, is an important chemotactic cytokine that modulates T-cell migration [35] and was found to be highly expressed (Fig 6T). *Rgs16*, which antagonizes inflammatory activation in monocytes [36], was significantly down-regulated (Fig 6U). *Gbp6*, an IFN inducible guanosine triphosphatase, was found to be highly expressed (Fig 6V). Altogether, pathway enrichment from each gene set revealed a molecular snapshot of the stages of microglial homeostatic restoration.

### NF-κB promotes microglia repopulation

**Fig 7:**
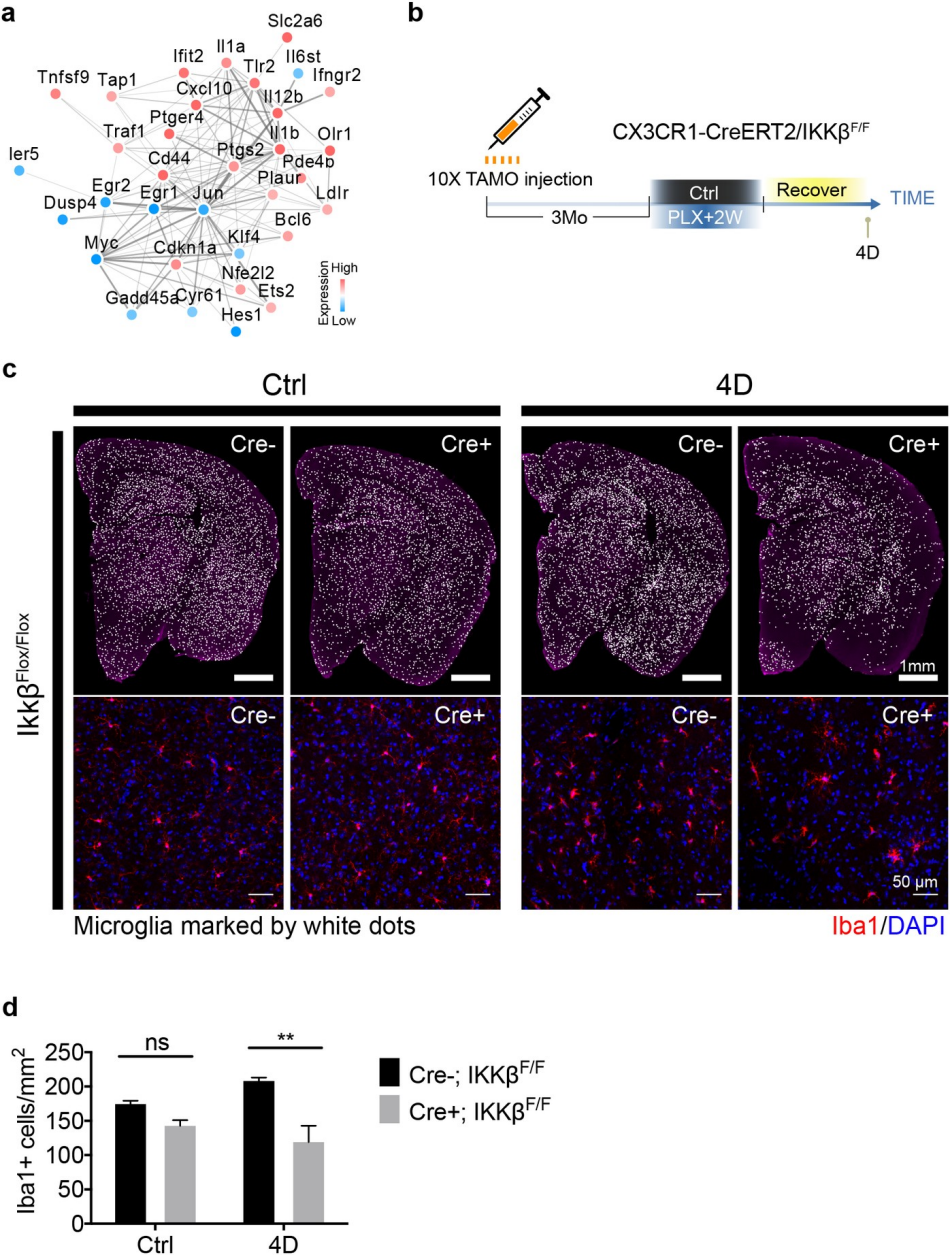
NF-kβ pathway promotes microglia repopulation. (a) Network of NF-kβ signaling–associated genes identified from the RNA-seq experiment (fast-return genes). Network was constructed using the STRING database. Directionality of gene expression pattern is shown as red for up-regulation and blue for down-regulation. (b) Experimental scheme for generating conditional IKKβ knockout mice. CX3CR1-CreERT2/IKKβ^f/f^ mice (7–8 Mo) injected with 2 mg tamoxifen/day via IP for 10 days. Three months later, mice were treated with PLX5622 diet for 2 weeks and switched to a normal diet for 4 D. (c) Representative images showing microglial density from the whole coronal section (upper panel) and thalamic region (lower panel). Iba1+ cells are visualized as white dots. (d) Quantification of microglial density from entire coronal section as shown in panel (c, upper panel) (mean ± SEM). Animal used: (*n* = 3) for each group. Two-way ANOVA with Sidak's multiple comparisons test was used to compare IKKβ control (Cre−) and IKKβ deletion (Cre+). *P* value is summarized as ns (*P* > 0.05), *(*P* ≤ 0.05), **(*P* ≤ 0.01), ***(*P* ≤ 0.001), and ****(*P* ≤ 0.0001). Individual numerical values can be found in S1 Data. CreERT2, tamoxifen-inducible Cre recombinase; CX3CR1, CX3C chemokine receptor 1; D, days; IKKβ, I-Kappa-B-Kinase Beta; IP, intraperitoneal; Mo, months; PLX, PLX5622; STRING, Search Tool for the Retrieval of Interacting Genes/Proteins.

Among the fast-returning genes, an intricate network of NF-κB pathway-associated genes was identified (Fig 7A). To understand the biological relationship between NF-κB signaling and early-phase microglial repopulation, we conditionally knocked out both copies of loxP-flanked I-Kappa-B-Kinase Beta (IKKβ) in microglia using CX3CR1-CreERT2/IKKβ^F/F^ mice (Fig 7B). This approach has been shown to suppress microglial activation mediated by the NF-κB pathway [37]. Conditional IKKβ knockout mice were treated for 2 weeks with PLX diet followed by 4 days of repopulation. Interestingly, IKKβ deletion showed a modest but statistically significant impairment in microglial repopulation density compared to Cre− control (Fig 7C and 7D). These results suggest that NF-κB signaling enhances the early phase of microglia repopulation.

### Cell death is associated with microglial proliferation

**Fig 8:**
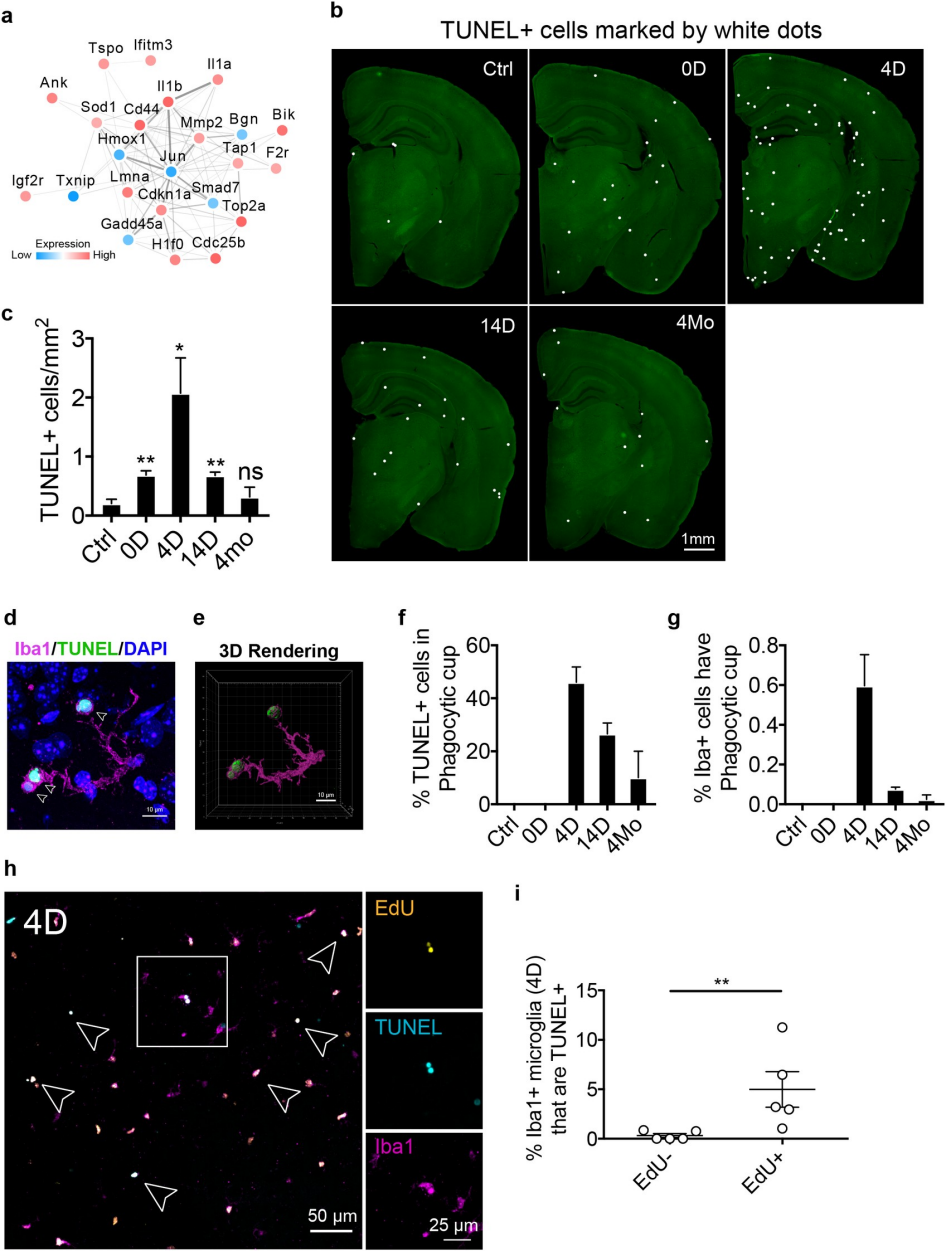
Cell death is associated with microglial proliferation. (a) Network of apoptosis-associated genes identified from the RNA-seq experiment (fast-return genes). Network was generated using the STRING database. Directionality of gene expression pattern is shown as red for up-regulation and blue for down-regulation. (b) Representative images of the coronal sections. C57/BL6J mice (3–5 Mo) were treated with PLX5622 diet for 2 weeks, then switched to control diet for various repopulation time points. TUNEL+ cells are marked with white dots. (c) Quantification of TUNEL+ cell density from entire coronal sections (mean ± SEM). Animal used: Ctrl (*n* = 5), 0 D (*n* = 6), 4 D (*n* = 5), 14 D (*n* = 7), and 4 Mo (*n* = 3). Statistical tests used: unpaired *t* test (Ctrl versus 0 D), unpaired *t* test with Welch's correction (Ctrl versus 4 D), Mann–Whitney test (Ctrl versus 14 D), and unpaired *t* test (Ctrl versus 4 Mo). (d) Example of microglia (4 D) forming phagocytic cup around TUNEL+ cells (highlighted by arrow heads). This image is rendered using max projection from a series of z-stack confocal images. (e) 3D model rendered from a series of z-stack confocal images. (f) Quantification of TUNEL+ cells that are in the phagocytic cup of Iba1+ microglia. (g) Quantification of Iba1+ microglia that exhibited phagocytic cup. (h) Representative confocal images of Iba1+ microglia, either EdU+ or EdU−, showing colocalization with TUNEL at day 4. EdU was given via IP injection every 24 hours for 4 days during repopulation. (i) Quantification of the percentage of TUNEL+ microglia (Iba1+) that are either EdU+ or EdU− (mean ± SEM). Animal used: 4 D (*n* = 5). Unpaired *t* test was used to compute statistical differences. *P* value is summarized as ns (*P* > 0.05), *(*P* ≤ 0.05), **(*P* ≤ 0.01), ***(*P* ≤ 0.001), and ****(*P* ≤ 0.0001). Individual numerical values can be found in S1 Data. Ctrl, control; D, days; EdU, 5-Ethynyl-2′-deoxyuridine; Iba1, ionized calcium binding adaptor molecule 1; IP, intraperitoneal; Mo, months; PLX, PLX5622; STRING, Search Tool for the Retrieval of Interacting Genes/Proteins.

Apoptosis-related genes were also highly represented among the fast-returning genes found in our RNA-seq data (Fig 6C and Fig 8A). To validate the involvement of cell death, we measured cell death by TUNEL assay at different stages of microglia repopulation. The number of TUNEL-positive cells was dramatically increased in the parenchyma, peaking at 4 days of repopulation (Fig 8B and 8C). This rate of death was significantly higher than at the end of PLX administration (0 D), suggesting that it was not directly caused by PLX treatment (Fig 8B and 8C). We also observed phagocytic cups, a special morphological feature that is indicative of phagocytosis [22, 38], around the TUNEL+ cells (Fig 8D and 8E). Further quantification in 4 D microglia show that approximately 40% TUNEL+ cells were in phagocytic cups (Fig 8F), while only 0.6% of microglia displayed this structure (Fig 8G), suggesting a high-clearance rate of dead cells in the repopulated brain and that the repopulated microglia are functionally intact. We next compared the percentage of TUNEL+ cells in 4 D microglia (EdU+Iba1+) versus resident microglia (EdU−Iba1+). We found that the TUNEL+ signal was more frequently associated with EdU+Iba1+ cells than EdU−Iba1+ cells (Fig 8H and 8I), suggesting that newborn microglia were more likely to undergo cell death. To rule out the possibility that the increased cell death might be an artifact of cell toxicity caused by EdU labeling, we compared the number of TUNEL+ cells in samples with or without EdU injection at 4 D and 14 D, which showed that EdU labeling did not skew the level of cell death (S10 Fig). Thus, active cell death is closely associated with microglial proliferation, consistent with findings by Askew and colleagues [11].

### Microglia homeostatic density is re-established through steady turnover

**Fig 9:**
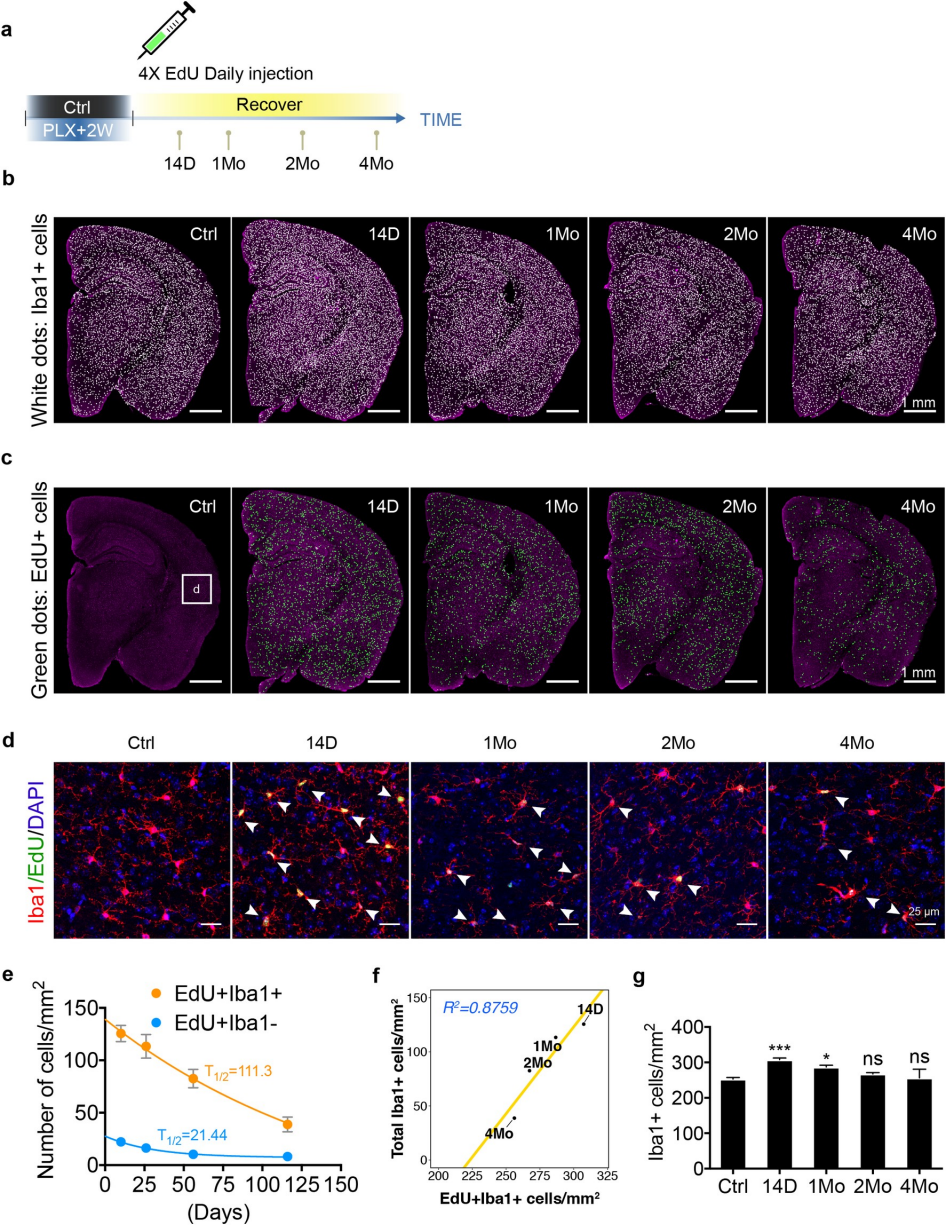
Homeostasis of adult newborn microglia is re-established through steady turnover. (a) EdU pulse-chase experiment to determine the longevity of newborn microglia. C57BL/6J mice (3–5 Mo) were treated with PLX5622 diet for 2 weeks and switched to a normal diet. EdU was injected via IP every 24 hours during the first 4 days of repopulation. Mice were analyzed after repopulation of 14 D, 1 Mo, 2 Mo, and 4 Mo. (b) Stitched coronal section showing microglia density. Iba1+ cells are visualized by white dots. (c) Stitched coronal section showing the density of EdU+ cells. EdU+ cells are visualized by green dots. (d) Zoomed in images from the cortical region (box “d” in panel c) showing colocalization of Iba1+ microglia (red) and EdU (green). Iba1+EdU+ cells are highlighted with white arrowheads. (e) Number of EdU+Iba1+ cells (orange line) and EdU+Iba1− cells (blue line) over time is fitted with nonlinear model: 1-phase exponential decay. Cell density (mean ± SEM) was quantified using the entire coronal area, as shown in panel c. T_1/2_ indicates half-life (days) for each population. Animals used: 14 D (*n* = 4), 1 Mo (*n* = 6), 2 Mo (*n* = 6), and 4 Mo (*n* = 3). (f) Linear regression showing the correlation between the decay of EdU+Iba1+ cells (x-axis) and the decay of total microglial cells (y-axis). R^2^ = 0.8579. (g) Quantification of Iba1+ microglial density using entire coronal area as shown in panel (b) (mean ± SEM). Animals used: 14 D (*n* = 4), 1 Mo (*n* = 6), 2 Mo (*n* = 6), and 4 Mo (*n* = 3). One-way ANOVA with Dunnett's multiple comparisons test was used by comparing to the Ctrl group. *P* value is summarized as ns (*P* > 0.05), *(*P* ≤ 0.05), **(*P* ≤ 0.01), ***(*P* ≤ 0.001), and ****(*P* ≤ 0.0001). Individual numerical values can be found in S1 Data. Ctrl, control; D, days; EdU, 5-Ethynyl-2′-deoxyuridine; Iba1, ionized calcium binding adaptor molecule 1; IP, intraperitoneal; Mo, months; PLX, PLX5622.

Interestingly, we and others have consistently observed an overproliferative phenotype that resembles microgliosis during the first week of repopulation (Fig 1B) [8–10]. We hypothesized that the resolution of microgliosis is achieved by egress of extra newborn cells. To measure the turnover rate of the repopulated microglia, we applied a pulse-chase approach using EdU labeling during the first 4 days of repopulation to track newborn microglia and extended the recovery time to 4 months (Fig 9A). As expected, the number of EdU+Iba1+ microglia decayed over time (Fig 9B, 9C and 9D). By fitting the data with a nonlinear 1-phase decay model, we estimated the half-life of repopulated microglia to be 111.3 days (Fig 9E). A much shorter lifespan was also determined for EdU+Iba1− cells (T_1/2_ = 21.44 days). The nonlinear 1-phase decay model assumes no additional proliferation, which was confirmed by the absence of proliferation marker Ki-67 after day 4 (S11 Fig). We found that the turnover rate of the EdU+Iba1+ microglia was well correlated with egress of excessive microglia (Fig 9F). Therefore, based on the decay rate of newborn microglia, microgliosis in the repopulated brain required up to 2 months to fully resolve (Fig 9G). Microgliosis resolution in the repopulated brain is also likely to be facilitated by microglia self-engulfment, as we observed examples of microglia forming phagocytic cups around dead microglia in both 14 D and 4 Mo brains (S12 Fig and S3–S6 Videos). Thus, restoration of microglial density seemed to involve steady turnover of newborn cells.

## Discussion

**Fig 10:**
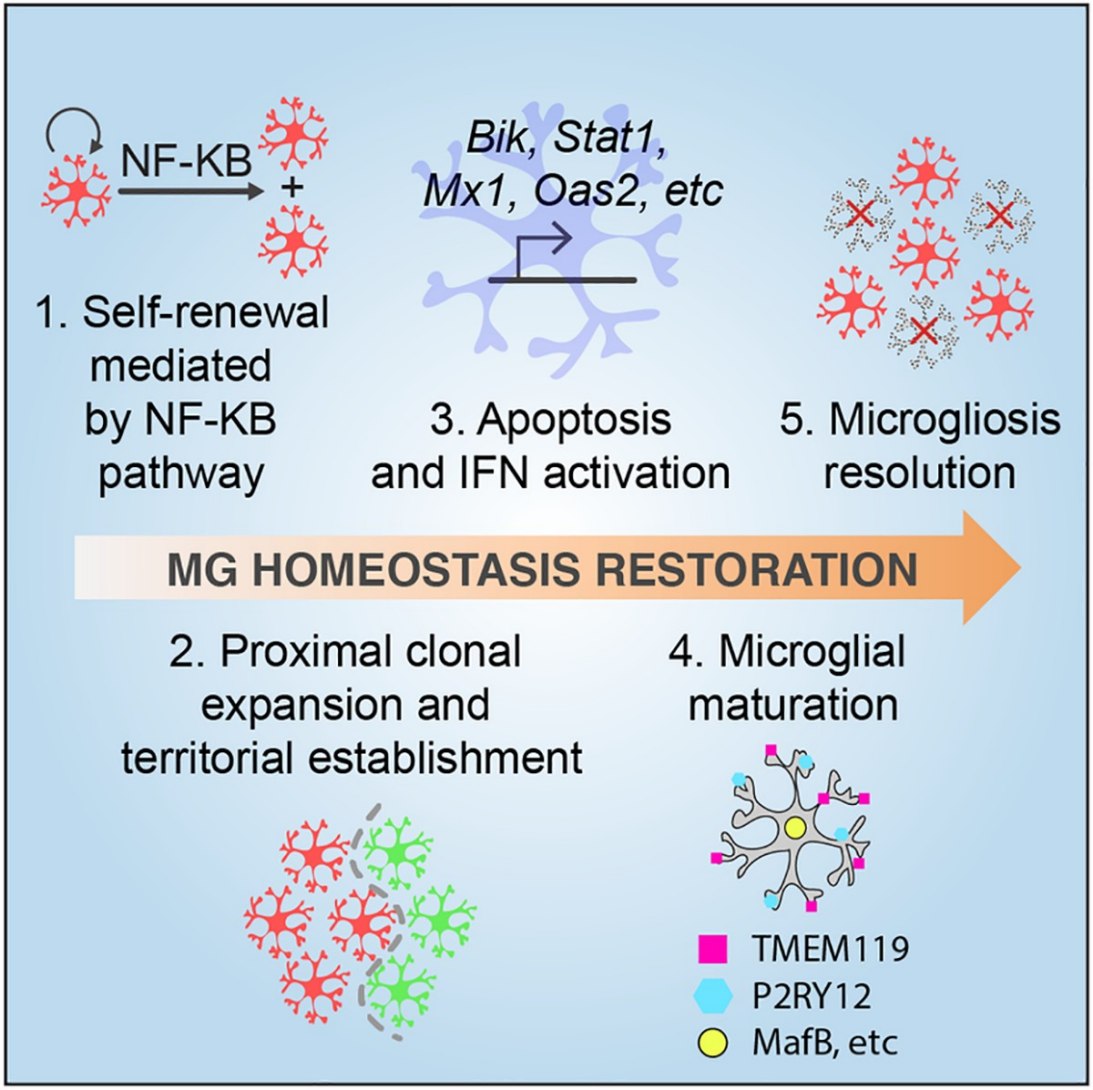
A model for the transitions from microgliogenesis to homeostatic establishment. Microglia are expanded through self-renewal. First, the proliferation of repopulating microglia partially requires the NF-κB pathway. Next, newborn microglia recolonize the parenchyma forming stable spatial clusters once the cell proliferation is completed. During this process, newborn microglia re-established maturity in a process that involves mitosis, apoptosis, and IFN pathway activation. Expression of mature markers such as P2RY12, TMEM119, and MafB is restored. Finally, microglia re-establish steady-state density through passive egress of excessive newborn cells. IFN, interferon; MafB, V-maf musculoaponeurotic fibrosarcoma oncogene homolog B; NF-κB, nuclear factor kappa-light-chain-enhancer of activated B cells; P2RY12, Purinergic Receptor P2Y12; TMEM119, Transmembrane Protein 119.

Our current study employed a chemical microglial ablation approach to query the mechanistic details of microglial homeostatic regulation. Remarkably, microglia seem to have inherent memory of their steady-state signature, as repopulated microglia eventually became almost identical to those in the resting state. The data presented here piece together a series of events that take place during microglial homeostatic restoration in adulthood (Fig 10). First, the depleted microglial pool expands through self-renewal that partially requires NF-κB signaling. Second, microglia recolonize the parenchyma from proximal clonal expansion and maintain stable spatial clusters. Third, newborn microglia re-establish maturity in a process that involves mitosis, apoptosis, interferon pathway activation, and surface molecule re-expression. Finally, homeostatic density is reached after egress of excessive newborn cells.

The existence of CNS-resident microglial precursors cells has been controversial [8]. In line with other studies [7, 9, 10, 39], our results demonstrate that the microglial pool can be replenished solely by self-renewal, with no detectable contribution from either resident CNS progenitors or peripheral circulating precursors. In the BMT chimera experiment, we observed most of the GFP+Iba1+ cells in the choroid plexus (Fig 2E), in line with recent findings that choroid plexus macrophages represent a distinctive parenchyma myeloid population that constantly receives cellular exchange with circulating monocytes [1]. Consistent with a previous report by Elmore and colleagues [8], we found a group of nonmicroglial cells that are highly proliferative after release from CSF1 inhibition (Fig 1D and S2 Fig), indicated by EdU labeling. Testing against a panel of common CNS markers, these cells appeared negative for Iba1, GFAP, NeuN, and S100β (Fig 1M). Although a small subset of them appeared to be DCX+ or Olig2+, the vast majority of cells may represent an uncharacterized cell type that remains to be further investigated. Nevertheless, our lineage-tracing experiments using CX3CR1-CreERT2 unambiguously demonstrate that this population does not represent microglial precursors.

Spatial characteristics of microgliogenesis were largely inaccessible in the past. Stochastic labeling in microglia using the Microfetti approach now opens new possibilities to extract spatial information during microglial tiling [22]. Using this approach, Tay and colleagues reported that microglia underwent clonal expansion in response to facial nerve injury [22]. In agreement with their finding, we found microglia also proliferate through clonal expansion following ablation. We also observed that not all seeding cells initiated clonal expansions, as evidenced by few scattered cells positioned between clusters (Fig 4C, 4D and 4F). This might reflect the heterogenous nature of microglia, arguing for the potential existence of a subpopulation of microglia with differential “stemness.” To examine the stability of microglial tiling, we followed the retiled parenchyma over the course of 1 month. We found that microglia only migrate during clonal expansion and that tiling boundaries remained stable up to 1 month, suggesting that once microglial expansion is completed, the tiling pattern appeared to be static.

Originating from yolk sac progenitors, microglia follow a series of transcriptional programs to reach maturity [25]. Recent studies have shown that adult microglial maturity is highly influenced by inflammatory signals [25, 40]. Exposure to immune-activating agents during pregnancy has also been shown to shift microglia to a more mature stage [25]. In contrast, adult microglia deprived of any immune stimuli (using a germ-free environment) display an immature morphology, immature transcriptional signatures, and a dampened response to lipopolysaccharides (LPS) challenge [25, 40]. These results suggest that microglial maturity is not a simple continuum of their birth age but rather is shaped by external factors such as immune activation.

In support of a role for immune signaling in maturation, we found that genes associated with the NF-κB pathway were highly enriched during the early phase of microglial repopulation (Fig 6C). Although the effects of microglia depletion under disease conditions have been investigated [41–43], how NF-κB activation in repopulating microglia would impact neuropathology is unknown and needs to be addressed in future studies. Inhibiting the NF-κB pathway via conditional knockout of IKKβ resulted in partially impaired microglial repopulation (Fig 7). Furthermore, we and others have found that adult microglial renewal is closely associated with cell death [11]. More studies are needed to clarify the biological significance for cell death during the maturation of newborn microglia.

Microglia have previously been considered a stable population [44], but this view has been recently challenged [11]. By quantifying the steady-state microglial proliferation rate, it was estimated that it takes 96 days to renew the entire rodent microglial pool [11]. However, a recent study by Tay and colleagues used a similar approach and concluded that microglia are extremely stable, with a steady-state turnover rate of over 41 months in the cortex, 15 months in the hippocampus, and 8 months in the olfactory bulb [22]. Although this study examined microglia in an unperturbed state, there are several caveats to its interpretation. First, assumptions regarding microglial cell cycle duration were made. Second, the labeling efficiency associated with nucleotide analogs such as EdU or Bromodeoxyuridine/5-bromo-2'-deoxyuridine (BrdU) might not capture all proliferating microglia, resulting in an underestimation of the turnover rate. Third, sampling power was greatly limited since only a handful of cells could be captured at a given time. To overcome these limitations, we used a pulse-chase strategy with EdU labeling to directly track the decay of repopulated microglia and showed that they have a half-life of 111.3 days. (Fig 9E). In other words, adult-born microglia have a lifespan of 7.5 months, such that a rodent brain could renew its entire microglial pool roughly 5 times in its lifetime. However, since our measurement started from repopulation day 14 when microglial homeostasis was not yet fully settled, 7.5 months may still be an underestimate of the actual microglial longevity. Indeed, a recent study by Füger and colleagues recorded microglial longevity via single-cell imaging and reported a lifespan of approximately 15 months [45]. In humans, microglial longevity is estimated be several decades long [46]. Thus, the egress of microglia that occurs following repopulation can be extremely slow and take months to fully resolve. In a way, this emphasizes the persistent nature of microgliosis in the context of sterile neuroinflammation. Therefore, strategies that can accelerate microglial turnover might be an overlooked avenue to treat neurological diseases for which microgliosis is the driving force.

The tremendous stability associated with microglial density over the lifetime in a rodent brain is quite remarkable [11]. Even after acute ablation, microglia are able to return to the same homeostatic density. The mechanisms by which microglia are able to remember their homeostatic density is still unclear. One possible way could be due to contact inhibition. After microglial ablation, residual microglia isolated in space would lose contact inhibition exerted by neighboring microglia, thus allowing them to freely multiply. The observed overproliferation (Fig 1A) [8–10] suggests that the inherent regulatory mechanism responsible for controlling density is switched off during microglial repopulation. Coincidentally, some surface molecules were still expressed at lower levels 1 month after repopulation including Syndecan-4, an important regulator of contact inhibition during cancer metastasis [34]. These genes are of great interest for future investigations (S1D Table). Nevertheless, the exact molecular machinery that sets the default homeostatic density remains to be characterized.

## Materials and methods

### Animals

All animal work was performed according to the approved guidelines from the University of California, San Francisco, Institutional Animal Care and Use Committee ID number (AN173162). Mice with ad libitum access to food and water were housed in a pathogen-free barrier facility with 12-hour light on/off cycle. C57BL/6J mice were used as wild-type controls. Equal numbers of male and female mice were used for all experiments except for the RNA-seq experiment, which used only female mice. Different mouse lines used in the study can be found in S2A Table.

### Drug administration

Diet containing 1,200 mg/kg PLX5622 (Plexxikon Inc., Berkeley) was given to mice as the sole food source for 2–3 weeks to deplete microglia. Control diet with the same base formula but without the compound was given to the control group. Tamoxifen (Sigma-Aldrich, T5648) was prepared in corn oil before use at 20 mg/mL. To efficiently induce Cre recombination, mice were given tamoxifen via intraperitoneal (IP) injection at 2 mg per day for 10 days. For sparse labeling in CX3CR1-CreERT2/Brainbow mice, tamoxifen was given via daily IP injection for 4 days. Mice receiving tamoxifen injections were housed for at least an additional 21 days after the last injection and before use. For labeling newborn cells, EdU (Santa Cruz, sc-284628) was prepared in sterile PBS at 20 mg/mL. EdU solution was warmed to 55°C before use to dissolve any chemical precipitation. Mice were injected at 80 mg/kg via IP diphtheria toxin (Sigma-Aldrich, D0564) and was resuspended in sterile PBS at 20 ug/mL working solution for injection. Diphtheria toxin was delivered to animals via IP injection at 1 ug per day for 3 days.

### BMT

Bone marrow was isolated from the tibias and femurs of 3–6 months old ACTB-eGFP transgenic mice. The bone marrow was triturated using an 18-gauge needle and passed through a 70-μm nylon mesh cell strainer to make a single-cell suspension. Erythrocytes were lysed with ACK lysis buffer (150 mM NH_4_Cl, 10 mM KHCO, and 0.1 mM Na_2_EDTA), washed with PBS, and suspended in PBS with 0.1% BSA at 3.5 × 10^7^ cells/mL. Recipient mice that were 4 to 6 months old were given 2 equal doses (2 × 600 rads, 3 hours apart) of irradiation with a cesium source irradiator. To protect the brain from radiation, the head of the mouse was shielded by a lead plate during irradiation. Irradiated mice were reconstituted with 7 × 10^7^ cells of donor bone marrow via tail vein injection. Repopulation efficiency was determined by counting the percentage of GFP-positive myeloid population (CD45+CD11b+) by flow cytometry.

### Flow cytometry

Blood and spleen samples were collected in EDTA. Erythrocytes were lysed in FCK lysis buffer for 5 min, and sample was pelleted at 500 g. Leukocytes were resuspended in FACS buffer (PBS, 0.5% BSA, 5% FBA, and 0.1% NaN3) and incubated for 15 min with anti-CD16/CD32 monoclonal antibodies (1:200, BD PharMingen) to block Fc receptors. Cells were then stained with APC-labeled anti-CD11b (1:100, Biolegend) and PE-labeled anti-CD45 (1:100, Biolegend) for 30 min on ice, followed by fixation with 2% PFA. Fluorescence intensity was measured using a FACS Calibur (BD Biosciences) flow cytometer. Data were analyzed in FlowJo (V10.0.7).

### MACS isolation of adult microglia

Adult microglia isolation was performed using MACS, as previously described [47]. Briefly, mice were anesthetized with avertin and transcardially thoroughly perfused with PBS to remove circulating blood cells in the CNS. Each dissected brain was chilled on ice and then minced in enzymatic digestion buffer containing 0.2% Collagenase Type 3 (Worthington, LS004182) and 3 U/mL Dispase (Worthington, LS02104). Minced brain tissue was then incubated at 37°C for 45 min. The enzymatic digestion was stopped with inactivation buffer containing 2.5 mM EDTA (Thermofisher, 15575020) and 1% fetal bovine serum (Invitrogen, 10082147). The digested brain tissue was then triturated in a serological pipette several times before passing through a 70-μm filter. The homogenate was then depleted of myelin using myelin removal beads (Miltenyi Biotec, 130-096-733) and magnetic LD column (Miltenyi Biotec, 130-042-901). The elute was enriched for microglia with CD11b magnetic beads (Miltenyi Biotec, 130-049-601) and MS column (Miltenyi Biotec, 130-042-201).

### Brain tissue harvest

Mice were anesthetized with avertin and transcardially perfused with PBS. Whole brains were drop-fixed in 4% paraformaldehyde prepared in PBS for 48 hours before switching to 30% sucrose for at least another 48 hours before cutting on a sliding microtome (Leica, SM2010R). Brain sections were prepared in a stereological manner. Sequential coronal planes in 30-μm thickness were collected and preserved in cyroprotectant containing 30% glycerol, 30% ethoxyethanol, and 40% PBS. Brain tissue samples were stored in −20°C before use. All staining experiments were performed on slices that were collected at the similar coronal planes.

### Immunohistochemistry

One or 2 coronal sections per mouse were used for each staining. Free-floating sections were washed in PBS and then permeabilized in PBST buffer (0.5% Triton X-100 diluted in PBS), followed by blocking in 3% normal donkey serum (NDS) at room temperature for 1 hour. Primary antibodies were diluted in PBST containing 3% NDS and incubated with tissues at 4°C overnight. Primary antibodies used in the study can be found in S2B Table. Secondary antibodies were then prepared the same way as primary antibodies and incubated with tissue at room temperature for 1 hour. All secondary antibodies were obtained from Jackson ImmunoResearch. Secondary antibodies used in the study can be found in S2B Table. After secondary antibody staining, DAPI nuclear stain was conducted during the washing step as needed. Tissues were then mounted on glass slides for further processing or applied with anti-fade mounting media (Vector Laboratories, H-1000) for imaging. For detecting EdU+ cells in brain sections, Click-iT EdU-imaging kits (ThermoFisher Scientific, C10337, C10339) were used following the manufacturer’s instructions before the immunofluorescence staining procedures. For terminal deoxynucleotidyl transferase-mediated dUTP nick end-labeling (TUNEL), the DeadEnd Fluorometric TUNEL System (Promega, G3250) was used with minor alterations to the manufacturer’s recommendations. We adapted a method developed by Deng and colleagues [48]. In brief, after the immunofluorescence staining was finished, free-floating brain sections were mounted on a charged glass slide (Fisher, 12-550-15) and dried at 55°C for 5 mins. Tissues were outlined with a hydrophobic pen (Vector Laboratories, H-4000). Mounted glass slides were then incubated in 0.5% Triton/PBS at 85°C for 20 min. After cooling to room temperature, the equilibration buffer from the kit was applied directly to the slide for 5 min, followed by application of TUNEL reaction solution and incubation at 37°C for 1 hour.

### Epifluorescence fluorescence microscopy

Regular epifluorescence images were acquired on a Keyence BZ-9000 inverted epifluorescence microscope equipped with an RGB and monochrome camera (Keyence, Osaka, Japan). Either the entire coronal hemibrain slice or particular brain regions were scanned using 10x magnification and stitched in Keyence BZ-X Analyzer software (V1.3.0.3).

### Confocal fluorescence microscopy

Confocal microscopy was performed with a Zeiss LSM880 inverted scanning confocal microscope (Carl Zeiss Microscopy, Thornwood, New York) equipped with 2 PMT detectors, a high-sensitivity GaAsP detector, and a 32-GaAsP Airyscan super-resolution detector and run by Zeiss Zen imaging software. Confocal fluorescence images were acquired with 5–10 focal planes at 1–2-μm intervals. Representative images are shown using Z-max intensity projections.

### Image analyses

All image analyses were performed in FIJI V1.50i [49]. Image analyses codes used are available on GitHub (https://github.com/lihong1github/Image-analysis). In brief, TIFF images were processed with adaptive threshold function to generate binary cell masks (https://sites.google.com/site/qingzongtseng/adaptivethreshold). Then, the “Analyze Particles” function was used for cell counting. Cell density (per mm^2^) was calculated by normalizing cell number to the size of the analyzed area. For analyzing cells with multiple markers, the region of interest (ROI) was first generated from the base channel. Channels containing other markers of interest were thresholded to generate binary images. Each ROI generated from the base channel was then overlaid on to the binary images to calculate percent area of the marker of interest. In general, >80% overlapping area is used to select ROIs that are positive for a second marker. To analyze microglia 3D morphology, confocal z-stacks with 8 focal planes of 2-μm interval were used. Three 425.1 × 425.1-μm^2^ connecting fields of view spanning the hippocampal CA1 to CA2 were captured and stitched in ZEN Blue software (Zeiss). The stitched tile images were analyzed in IMARIS software (V9.0.2, Bitplane). Microglia processes were analyzed using IMARIS filament function. Microglia soma size were measured using IMARIS surface function.

### Acute brain slice imaging

Heterozygous CX3CR1^eGFP/+^ mice of 3–3.5 months old were treated for 2 weeks with a PLX-containing diet and then switched to a regular diet to allow microglial repopulation for 6 days. Acute brain slices were prepared, as previously described [47]. Mice were anesthetized with isoflurane and perfused with 20 mL of ice-cold, carbogen-saturated (95% O_2_, 5% CO_2_) NMDG-HEPES artificial cerebrospinal fluid (ACSF) solution containing 93 mM NMDG, 2.5 mM KCl, 1.4 mM NaH_2_PO_4_, 30 mM NaHCO_3_, 20 mM HEPES, 25 mM D-glucose, 5 mM ascorbic acid, 2 mM thiourea, 3 mM sodium pyruvate, 12 mM N-acetyl-L-cysteine, 10 mM MgSO_4_, and 0.5 mM CaCl_2_. The ACSF was adjusted to pH 7.4 before use. All reagents were obtained from Sigma. Brains were washed in NMDG-HEPES. Coronal slices (300-μm thick) were prepared using a vibratome (Microm, Walldorf, Germany) at 4°C. For imaging, hemibrain slices were transferred to a 35-mm glass bottom dish (MatTek) and secured under a slice anchor (Warner instruments). Imaging sessions were conducted within 4 hours after decapitation and preparation of the slice in order to reduce expression of activation markers by microglia. All imaging was done in non-NMDG ACSF solution containing (in mM) NaCl 92, KCl 2.5, NaH2PO4 1.4, NaHCO3 30, HEPES 20, D-glucose 25, Ascorbic acid 5, Thiourea 2, Sodium pyruvate 3, N-acetyl-L-cysteine 12, MgSO4 2, CaCl2 2, and pH 7.4 (all from Sigma). Carbogen-saturated ACSF flowed continuously over the slice using a perfusion pump system (Cole-Parmer) at a rate of 3 mL/min. Images were captured with a Zeiss Z1 Observer inverted epifluorescence microscope (Carl Zeiss Microscopy, Thornwood, New York) with an ORCA-Flash 4.0 sCMOS camera (Hamamatsu Photonics, Shizuoka, Japan) and a Zeiss Axiocam MRm monochrome camera run by Zeiss Zen imaging software. The microscope is equipped with a motorized stage and temperature-controlled incubation system. Acquisition of cortical microglia was performed at a range of 50 to 100 μm from the surface of the slice to prevent capture of activated microglia, and z-stack images (1-μm step-size) were taken every 60 seconds for 15 to 30 min at 488-nm excitation and 510-nm emission wavelengths using a 40x objective. The motility of microglia processes was analyzed using the ImageJ plugin MTrackJ [50] to calculate the velocity of process retraction and extension per imaged cell.

### Microglia spatial pattern analyses

Hemibrain slices from CX3CR1-CreERT2/Brainbow mice were stained with anti-GFP and anti-RFP antibody. The entire coronal section was scanned using a Keyence BZ-9000 inverted epifluorescence microscope. RFP+ or GFP+ cells were segmented, and the XY coordinates were extracted using centeroids function in FIJI. NNDs were calculated with ImageJ plugin NND (https://icme.hpc.msstate.edu/mediawiki/index.php/Nearest_Neighbor_Distances_Calculation_with_ImageJ). NND of the labeled cells was extracted from an entire hemibrain coronal slice with area of 25.5 ± 1.03 mm^2^. Spatial point pattern clustering analysis with Ripley’s K-function was performed, as previously described [51]. The K-function is expressed as Eq 1, in which *n* is the number of total cells, *r* is the varying radius, *N*_*Pi*_*(r)* is the regional density of the *i*th cell at radius r, and *λ* is overall cell density. The overall degree of “Clusterness” is estimated as a cumulative function of the radius *r*. A negative score in H-function indicates patterns of dispersion, while a positive score indicates patterns of clustering. Theoretical complete spatial randomness (CSR) modeled by Poisson distribution in K(r) equals πr^2^. H-function, as shown in Eq 2, is transformed from the Ripley’s K-function for improved data visualization. In H-function, CSR equals zero.

$$K\left(r\right)=\frac{1}{n}\sum_{i=1}^{n}\frac{N_{Pi}\left(r\right)}{\lambda }$$ (1)

$$H\left(r\right)={\left(\frac{K\left(r\right)}{\pi }\right)}^{1/2}-r$$ (2)

H(r) function was computed using Kest function in R package Spatstat [52]. H(r) values from each mouse were calculated separately. Average H(r) were then computed for each treatment group. Smoothing function using a moving average with interval of [*r* − 15 μm, *r* + 15 μm] was applied. Domain size was estimated from the corresponding radius at maximum of H(r) [24]. To define cluster boundaries, 2D kernel density estimates were calculated by using density function in Spatstat package. Spatial points of RFP+ or GFP+ cells were smoothed with 100 μm^2^ Gaussian kernel to generate density estimates. Regions containing the highest top 10% kernel density were chosen to generate cluster boundaries, as this parameter best matches expected cell density. Overall size shift of the RFP+/GFP+ cluster overlapping region were quantified as percent of RFP and GFP overlapping area relative to the combined nonoverlapping RFP and GFP area.

### Quantitative PCR

RNA was extracted using Direct-zol RNA micro-prep kit (Zymo Research, R2061) following the manufacturer’s instructions. Roughly 100,000 microglia cells were lysed in 300 μl of Direct-zol reagent. Genomic DNA digestion was performed on column using DNaseI (Zymo Research, E1010). Extracted RNA (approximately 500 ng) was then converted to cDNA using iScript cDNA synthesis kit (Bio-Rad, 1708890) and diluted 10-fold with nuclease free water as template DNA. qPCR was performed using SYBR Green PCR Master Mix (Applied Biosystems, 4309155). Data were acquired using 7900HT real-time system equipped with a 384-well thermal block (Applied Biosystems, Forster City, United States of America). Raw Ct values were obtained using the Sequence Detection Systems software (Applied Biosystems, version 2.4). Relative gene expression was calculated based on the delta–delta Ct method using qbase+ software (Biogazelle, version 3.0). GAPDH was used as reference gene. Primer sequences can be found in S2C Table.

### High-throughput RNA sequencing

Female wild-type C57BL6 mice of 5–6 months were used. To identify differential gene expression during microglial repopulation, mice were treated for 2 weeks with PLX-containing diet and then switched to regular diet allowing microglial repopulation for various durations before being sacrificed for microglia isolation. These repopulation duration time points were set at 4 days (4 D), 14 days (14 D), and 1 month (1 Mo). P4 microglia were isolated from 4-day-old postnatal mice as an immature microglia control. Microglia isolated from each single mouse were used as an individual sample for downstream steps. A total of 3 pups (P4), 4 untreated adult mice (Ctrl), and 4 repopulated mice from each designed time point were used. Total RNA from fresh isolated microglia was extracted using Direct-zol RNA microprep kit (Zymo Research, R2061). cDNA library generation and RNA-seq service was performed by Novogene (Novogene Co., Ltd, Sacramento, California). RNA quality was examined by Bioanalyzer 2100 (Agilent Genomics). RNA samples with RIN value greater than 8 were used for cDNA library. Oligo(dT) beads were used to enrich for mRNA. After chemical fragmentation, a cDNA library was generated using NEBNext Ultra RNA Library Prep Kit for Illumina (New England Biolabs, E7530S). Quality of the cDNA library was assessed using Qubit assay for preliminary concentration, Bioanalyzer 2100 for insert size, and qPCR for effective library size. QC passed cDNA library samples were then sequenced with the HiSeq 4000 system (Illumina) at PE150. On average, less than 0.01% error rate was detected, and over 95% effective rate was observed in all sequencing results. Raw read ends containing low-quality reading or adapter sequence were trimmed prior to downstream analysis.

### RNA-seq data analyses

RNA-seq read mapping was performed using the STAR program [53]. Gencode mouse genome GRCm38 was used as reference (release M16, 2017). On average, roughly 80% of reads were uniquely mapped to the reference genome. The read count table was generated with the RSEM program [54]. Differential gene expression was calculated with R package edgeR [55] and limma [56]. Genes which showed less than 1 count per million (CPM) in at least 3 samples were filtered out from further analysis. Normalization was performed with the weighted trimmed mean of M-values (TMM) method [57]. RNA-seq data were then transformed for linear model fitting with voom and lmFit functions inside the limma package. Finally, empirical Bayes statistics were applied to correct variance of genes with low expression in the data set. FDR was calculated by the Benjamini–Hochberg method [58]. DE genes were defined as 2-fold change with FDR less than 0.05 in comparison to unperturbed adult microglia (Ctrl).

### Gene network and functional analyses

Gene network analyses were performed with GSEA with molecular signatures database (MSigDB) [30, 31]. Network visualization was made in Cyotscape (version 3.6.0) with access to STRING database [59].

### Statistics

All experiments were performed with a minimum of at least 3 biological replicates. All data were averaged to individual animals. Mean values from each animal were used for computing statistical differences. Error bar in plots indicates standard error of the mean (SEM). Statistical analyses were performed in Graphpad prism 7.0c (Graphpad, San Diego, California) and R (F Foundation for Statistical Computing, Vienna, Austria). Data visualization were achieved with R package ggplot2 [60]. Data normality was assessed using the Shapiro–Wilk normality test. F test was used to assess homoscedasticity prior to unpaired *t* test, and Brown–Forsythe test was used to homoscedasticity prior to ANOVA. For data with normal distribution and equal variance, unpaired *t* test was used to compare 2 groups. One-way ANOVA was used to compare data with more than 2 groups. Dunnett's multiple comparisons test was used to compare difference between designated groups. Two-way ANOVA was used for groups with genotypes and treatment as factors. Sidak's multiple comparisons test was used to compare statistical difference between genotypes. For data that failed to pass the normality test, Mann–Whitney test (for 2 groups) and Kruskal–Wallis test (>2 groups) were applied. For data with unequal variance, unpaired *t* test with Welch's correction were applied. *P* value and FDR are summarized as ns (*P* > 0.05), *(*P* ≤ 0.05), **(*P* ≤ 0.01),***(*P* ≤ 0.001), and ****(*P* ≤ 0.0001).

## Supporting information

S1 Fig Microglial repopulation occurs after DT-mediated microglial depletion. (a) Experimental scheme of DT-mediated microglial depletion and repopulation. Tamoxifen was given via IP injection to CX3CR1-CreERT2/STOP^flox^-DTR mice (7–8 Mo) to induce expression of DT receptor on microglia. DTX was injected IP every 24 hours for 3 days to deplete microglia. Mice were analyzed 1 day (DT + 1 D) or 7 days (DT + 7 D) after the last DT injection. (b) Representative images showing Iba1+ microglia (red) density. (c) Quantification of Iba1+ microglial density using entire coronal section (mean ± SEM). Animals used: Ctrl (*n* = 4); DT + 1 D (*n* = 6); DT + 7 D (*n* = 6). One-way ANOVA with Dunnett's multiple comparisons test was used to compare with Ctrl group. P value is summarized as ns (*P* > 0.05); *(*P* ≤ 0.05); **(*P* ≤ 0.01); ***(*P* ≤ 0.001); ****(*P* ≤ 0.0001). Individual numerical values can be found in S1 Data. CreERT2, tamoxifen-inducible Cre recombinase; Ctrl, control; CX3CR1, CX3C chemokine receptor 1; D, days; DT, diphtheria toxin; Iba1, ionized calcium binding adaptor molecule 1; IP, intraperitoneal; Mo, months.
(TIF)

S2 Fig EdU labeling during microglial repopulation at day 4. Confocal microscopy images showing microglial depletion and repopulation in different brain regions. The following markers were pseudo-colored: Iba1 (red), EdU (green), and DAPI (blue). DAPI, 4′,6-diamidino-2-phenylindole; EdU, 5-Ethynyl-2′-deoxyuridine; Iba1, ionized calcium binding adaptor molecule 1.
(TIF)

S3 Fig Increased microglial movement at 6 D of repopulation. (a, b) Representative frames from live imaging of untreated control microglia (b) and microglia at day 6 of repopulation (c). Acute slices from CX3CR1^eGFP/+^ mice were used to image microglia. A total of 16 mins were recorded. The first frame (pseudo-colored in red) is overlaid with the last frame (pseudo-colored in green). The box highlights movement of microglial processes. Extension is indicated with closed triangles, while retraction is indicated with open triangles. (c) Quantification of the average velocity of all processes per cell in μm/sec from acute brain slices (mean ± SEM). Ctrl (*n* = 3 animals, 6 slices, 26 cells); 6 D (*n* = 2 animals, 10 slices, 42 cells). Data from each cell are plotted. Unpaired *t* test was applied. *P* value is summarized as ns (*P* > 0.05); *(*P* ≤ 0.05); **(*P* ≤ 0.01); ***(*P* ≤ 0.001); ****(*P* ≤ 0.0001). Individual numerical values can be found in S1 Data. CX3CR1^eGFP^, microglia reporter line expresses eGFP under CX3CR1 promoter; Ctrl, control; D, days.
(TIF)

S4 Fig BMT reconstituted peripheral monocytes in the recipient mice. (a) Samples of the blood and spleen homogenate from the BMT mice were analyzed with FACS. Representative FACS gating plots from spleen samples are shown here. The monocytic population was selected by CD45 and CD11b and immunopositivity. Detailed gating strategy can be found in S3 Data. (b) GFP+ cells in the myeloid population were further separated and compared with the non-BMT Ctrl. (c) Quantification of bone marrow reconstitution efficiency in BMT mice. Reconstitution efficiency was defined as the percentage of GFP+CD45+CD11b+ cells out of all the CD45+CD11b+ cells. Animals used: 14 D (*n* = 5) and 2 Mo (*n* = 5). Individual numerical values can be found in S1 Data. BMT, bone marrow transplantation; CD, cluster of differentiation; Ctrl, control; D, days; FACS, fluorescence activated cell sorting; GFP, green fluorescent protein; Mo, months.
(TIF)

S5 Fig PDGFra+ and NG2+ precursor cells do not contribute to adult microglial repopulation. (a) Representative images of microglial depletion (PLX treatment for 2 weeks) and repopulation (normal diet for 1 week) in PDGFra-CreERT2/STOP-^flox-^RFP mice. Microglia are labeled with Iba1 (green). Progenitor cells from PDGFra lineage are labeled with RFP (red). (b–d) Analysis of PDGFra-CreERT2/STOP-^flox-^RFP mice before and after microglia repopulation. Quantification of Iba1+ microglia density (b), RFP+ cell density (c), and percentage of microglia that express RFP (d) are shown (mean ± SEM). Animals used: Ctrl (*n* = 3); Del (*n* = 3); Repop (*n* = 4). Kruskal–Wallis test was used for b. One-way ANOVA was used for c. (e) Representative images of microglial depletion (PLX treatment for 2 weeks) and repopulation (normal diet for 1 week) in NG2-CreERT2/STOP-^flox-^RFP mice. Microglia are labeled with Iba1 (green). Progenitor cells from NG2 lineage are labeled with RFP (red). (f–h) Analysis of NG2-CreERT2/STOP-^flox-^RFP mice before and after microglial repopulation. Quantification of Iba1+ microglia density (f), RFP+ cell density (g), and percentage of microglia that express RFP (h) are shown (mean ± SEM). Animals used: Ctrl (*n* = 3); Del (*n* = 4); Repop (*n* = 5). One-way ANOVA was used for statistical test. *P* value is summarized as ns (*P* > 0.05); *(*P* ≤ 0.05); **(*P* ≤ 0.01); ***(*P* ≤ 0.001); ****(*P* ≤ 0.0001). Individual numerical values can be found in S1 Data. CreERT2, tamoxifen-inducible Cre recombinase; Ctrl, control; Del, deletion; Iba1, ionized calcium binding adaptor molecule 1; NG2; neural/glial antigen 2, PDGFra, platelet derived growth factor receptor alpha; PLX, PLX5622; Repop, repopulation; RFP, red fluorescent protein.
(TIF)

S6 Fig Iba1 count and NND analysis of Iba1+ cells from CX3CR1-CreERT2/Brainbow mice. (a) Representative images showing microglial density before and after repopulation in CX3CR1-CreERT2/Brainbow mice. Mice were administered PLX5622 diet for 2 weeks before switching to normal diet and were sacrificed at various time points. Images were taken from the thalamic region. (b, c) Quantification of Iba1+ microglial density (b) and the NND of all Iba1+ microglia (c) (mean ± SEM). Animals used: Ctrl (*n* = 5), 0 D (*n* = 3), 7 D (*n* = 4), and 1 Mo (*n* = 5). Individual numerical values can be found in S1 Data. CreERT2, tamoxifen-inducible Cre recombinase; Ctrl, control; CX3CR1, CX3C chemokine receptor 1; D, days; Iba1, ionized calcium binding adaptor molecule 1; Mo, months; NND, nearest neighbor distance; PLX, PLX5622.
(TIF)

S7 Fig Microglia purity and validation for RNA-seq. (a) Bar graph showing the presence of markers associated with major CNS cell types in the RNA-seq data. Mean value of TPM was plotted. (b) Relative expression level of selected genes from the RNA-seq data (14 D). Animals used: Ctrl (*n* = 4) and 14 D (*n* = 4). Yellow line (y = 1) represents expression level of control. (c) Relative expression level of selected genes validated by qPCR. Animal used: Ctrl (*n* = 11) and 7 D (*n* = 12). Yellow line (y = 1) represents expression level of control. Unpaired *t* test was used. (d) Linear regression showing the correlation between the RNA-seq data from 14 D microglia and the qPCR data from 7 D microglia (R^2^ = 0.8494). *P* value is summarized as ns (*P* > 0.05), *(*P* ≤ 0.05), **(*P* ≤ 0.01), ***(*P* ≤ 0.001), ****(*P* ≤ 0.0001). Individual numerical values can be found in S1 Data. CNS, central nervous system; Ctrl, control; D, days; qPCR, quantitative PCR; TPM, transcript per million.
(TIF)

S8 Fig 4 D adult newborn microglia share similar transcriptome signature with neonatal P4 microglia. (a) Poission distance matrix showing relative similarity between each treatment group. Average Poission distance values from biological replicates were used. Relative Poission distance score is shown, which has a range of 0 (no similarity) to 100 (completely same). (b) KEGG pathway analysis using up-regulated genes found in both P4 neonatal and 4 D repopulated microglia. Top-10 enriched KEGG terms are shown. (c) Network showing cell cycle–related genes identified in pane (b). Network was constructed using STRING database. (d) KEGG pathway analysis using down-regulated genes found in both P4 and 4 D microglia. Top-10 enriched KEGG terms are shown. (e) Network showing MAPK and TGF-β signaling–related genes identified in panel (d). Network was constructed using STRING database. D, days; KEGG, Kyoto Encyclopedia of Genes and Genomes; MAPK, Mitogen Activated Protein Kinase; TGF-β, transforming growth factor β; STRING, Search Tool for the Retrieval of Interacting Genes/Proteins.
(TIF)

S9 Fig Adult newborn microglia re-establish static maturity over time. (a) Representative confocal images showing morphological features of microglia at different repopulation stages (upper) and microglia processes rendered from 3D model (lower). C57/BL6J cice (5 Mo) were treated with PLX5622 diet for 2 weeks and then switched to a normal diet at indicated time points. (b, c, d) Quantification of the number of microglia process terminal points (b), total length of the processes (c), and soma size (d). Greater than 150 microglial cells from each animal were analyzed. Mean value from each animal was plotted with SEM. Animals used: Ctrl (*n* = 5), 4 D (*n* = 5), 14 D (*n* = 4), 1 Mo (*n* = 5), and 4 Mo (*n* = 3). (e, f) Representative confocal images showing expression of P2RY12 (e) and TMEM119 (f) at different repopulation stages. Images were taken from the hippocampal region. (g, h) Quantification of P2RY12 (g) and TMEM119 (h) positive area per Iba1+ microglia cell number (mean ± SEM). Animals used: Ctrl (*n* = 5), 4 D (*n* = 5), 14 D (*n* = 4), 1 Mo (*n* = 5), and 4 Mo (*n* = 3). Statistical analyses: 1-way ANOVA with Dunnett's multiple comparisons test (against Ctrl) was used in panel (b, c); Kruskal–Wallis test with Dunnett's multiple comparisons test (against Ctrl) was used in panel (d, g, h). Individual numerical values can be found in S1 Data. Ctrl, control; D, days; Mo, months; P2RY12, Purinergic Receptor P2Y12; PLX, PLX5622; TMEM119, Transmembrane Protein 119.
(TIF)

S10 Fig EdU labeling does not affect microglial apoptosis. (a) Quantification of TUNEL+ cell density with or without EdU injection. EdU was injected every 24 hours via IP for 4 days of repopulation (mean ± SEM). Number of animals used for 4 D group: Ctrl (*n* = 5), EdU+ (*n* = 5). Unpaired *t* test was used. (b) Same analysis as in (a) with extended repopulation to 14 days. Number of animals used: Ctrl (*n* = 3), EdU+ (*n* = 4). Mann–Whitney test was used. Individual numerical values can be found in S1 Data. Ctrl, control; D, days; EdU, 5-Ethynyl-2′-deoxyuridine; IP, intraperitoneal.
(TIF)

S11 Fig Adult newborn proliferation subsides after 2 weeks. Representative confocal images showing Ki-67 (yellow) and Iba1 (magenta) staining in mice used to trace newborn microglial turnover. Images were taken from the amygdala. Only repopulation at 4 D shows noticeable Ki-67 positive staining. D, days; Iba1, ionized calcium binding adaptor molecule 1; Ki-67, Proliferation marker protein Ki-67.
(TIF)

S12 Fig Apoptotic and phagocytic microglia during microgliosis resolution. Representative confocal images showing TUNEL+ microglia and microglia forming phagocytic cup around TUNEL+ cells at either 14 D or 4 Mo. D, days; Mo, months.
(TIF)

S1 Video Movement of control microglia. (AVI)

S2 Video Movement of repopulated microglia (6 D). (S1 and S2 Videos) Projection images were created with stacks of fluorescent images acquired during time-lapse imaging of acute brain slices (1 min sampling interval, 2-μm z-stacks, rate 5 frame per second, time stamp in mins, scale bar = 10 μm).
(AVI)

S3 Video 3D reconstruction of TUNEL+ microglia after 14 days of repopulation. (MOV)

S4 Video 3D reconstruction of TUNEL+ microglia after 4 months of repopulation. (MOV)

S5 Video 3D reconstruction of 14 D microglia phagocytosing another TUNEL+ microglia. (MOV)

S6 Video 3D reconstruction of 4 Mo microglia phagocytosing another TUNEL+ microglia. (S3–S6 Videos) Iba1 (red), TUNEL (green), and DAPI (blue). DAPI, 4′,6-diamidino-2-phenylindole; Iba1, ionized calcium binding adaptor molecule 1; Mo, months.
(MOV)

S1 Table DE gene list of RNA-seq. (a) All genes. (b) Fast-return genes. (c) Medium-return genes. (d) Slow-return genes. (e) Delayed-response genes. DE, differentially expressed.
(XLS)

S2 Table Mouse line, antibodies, and primers. (a) Mice used in the study. (b) Antibodies list. (c) qPCR primers. qPCR, quantitative PCR.
(XLS)

S1 Data Numerical data and statistical analyses. Numeric data shown in separate excel spreadsheets.
(XLS)

S2 Data RNA-seq data. (a) Raw read counts. (b) TPM. TPM, transcript per million.
(XLS)

S3 Data FACS gating strategy used in BMT experiment. BMT, bone marrow transplantation; FACS, fluorescence-activated cell sorting.
(TIF)

## Abbreviations

BMT: bone marrow transplantation
BrdU: Bromodeoxyuridine/5-bromo-2'-deoxyuridine
CNS: central nervous system
CreERT2: tamoxifen-inducible Cre recombinase
CSF1R: colony stimulating factor 1 receptor
CX3CR1: CX3C chemokine receptor 1
CFP: cyan fluorescent protein
DAPI: 4′,6-diamidino-2-phenylindole
DCX: doublecortin
DE: differentially expressed
EdU: 5-Ethynyl-2′-deoxyuridine
FACS: fluorescence-activated cell sorting
FDR: false discovery rate
GFP: green fluorescent protein
GSEA: gene set enrichment analysis
Iba1+: ionized calcium binding adaptor molecule 1
IKKβ: I-kappa-b-kinase beta
IFN: interferon
LPS: lipopolysaccharides
MACS: magnetic-activated cell sorting
MAPK: mitogen activated protein kinase
MSigDB: molecular signatures database
NF-κB: nuclear factor kappa-light-chain-enhancer of activated B cells
NG2: neural/glial antigen 2
NND: nearest neighbor distance
Olig2: oligodendrocyte transcription factor 2
OPC: oligodendrocyte precursor cell
PCA: principal component analysis
PDGFra: platelet derived growth factor receptor alpha
PLX: PLX5622
qPCR: quantitative PCR
RFP: red fluorescent protein
SGC: subgranular zone
TGF-β: transforming growth factor β
TPM: transcript per million
YFP: yellow fluorescent protein
