# Supplementary figures and images for: Proximal recolonization by self-renewing microglia re-establishes microglial homeostasis in the adult mouse brain
Source: PLoS Biol. 2019 Feb 8;17(2):e3000134. doi: 10.1371/journal.pbio.3000134 (PMC6383943; doi:10.1371/journal.pbio.3000134)

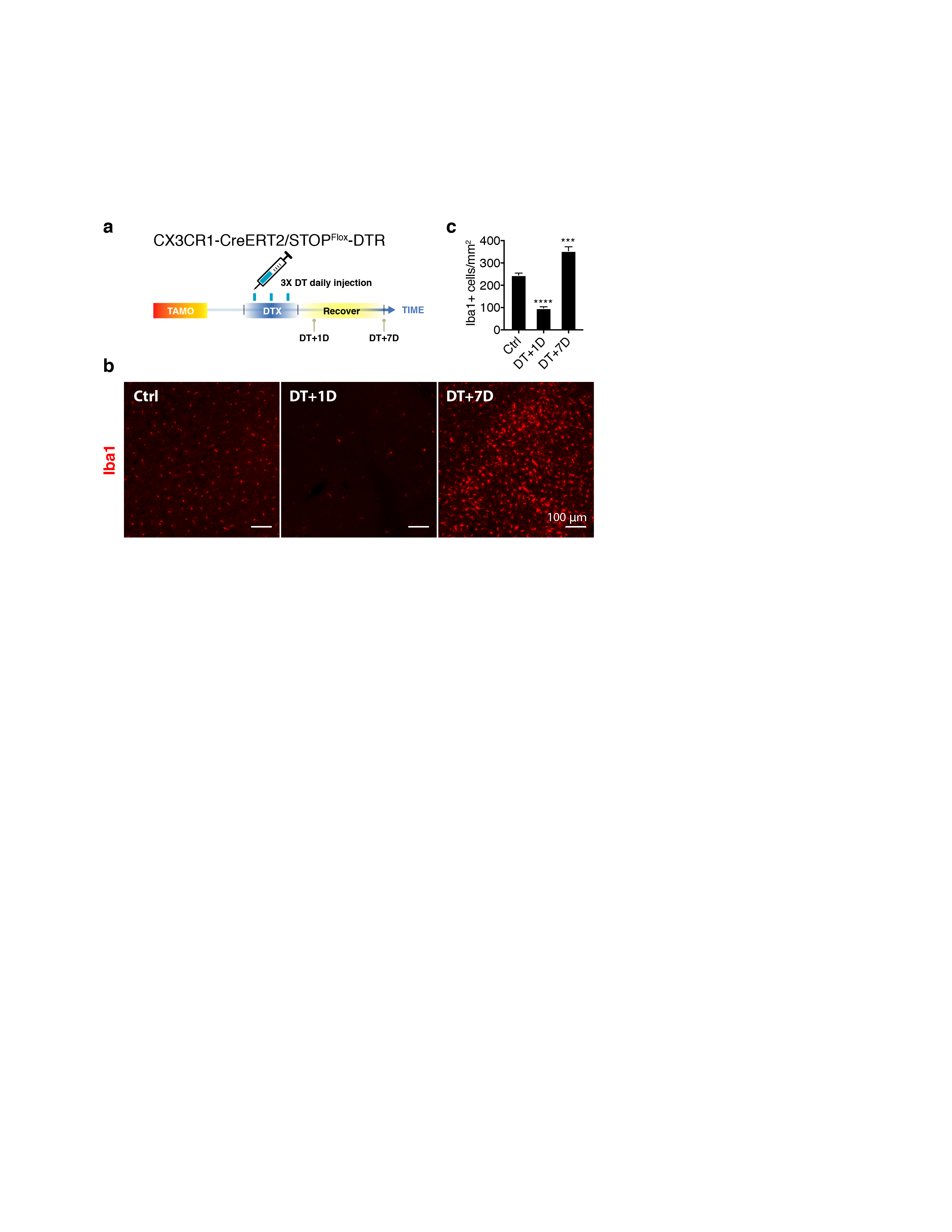

Supplement: S1 Fig — (a) Experimental scheme of DT-mediated microglial depletion and repopulation. Tamoxifen was given via IP injection to CX3CR1-CreERT2/STOPflox-DTR mice (7–8 Mo) to induce expression of DT receptor on microglia. DTX was injected IP every 24 hours for 3 days to deplete microglia. Mice were analyzed 1 day (DT + 1 D) or 7 days (DT + 7 D) after the last DT injection. (b) Representative images showing Iba1+ microglia (red) density. (c) Quantification of Iba1+ microglial density using entire coronal section (mean ± SEM). Animals used: Ctrl (n = 4); DT + 1 D (n = 6); DT + 7 D (n = 6). One-way ANOVA with Dunnett's multiple comparisons test was used to compare with Ctrl group. P value is summarized as ns (P > 0.05); *(P ≤ 0.05); **(P ≤ 0.01); ***(P ≤ 0.001); ****(P ≤ 0.0001). Individual numerical values can be found in S1 Data. CreERT2, tamoxifen-inducible Cre recombinase; Ctrl, control; CX3CR1, CX3C chemokine receptor 1; D, days; DT, diphtheria toxin; Iba1, ionized calcium binding adaptor molecule 1; IP, intraperitoneal; Mo, months. (TIF) [file pbio.3000134.s001.tif]

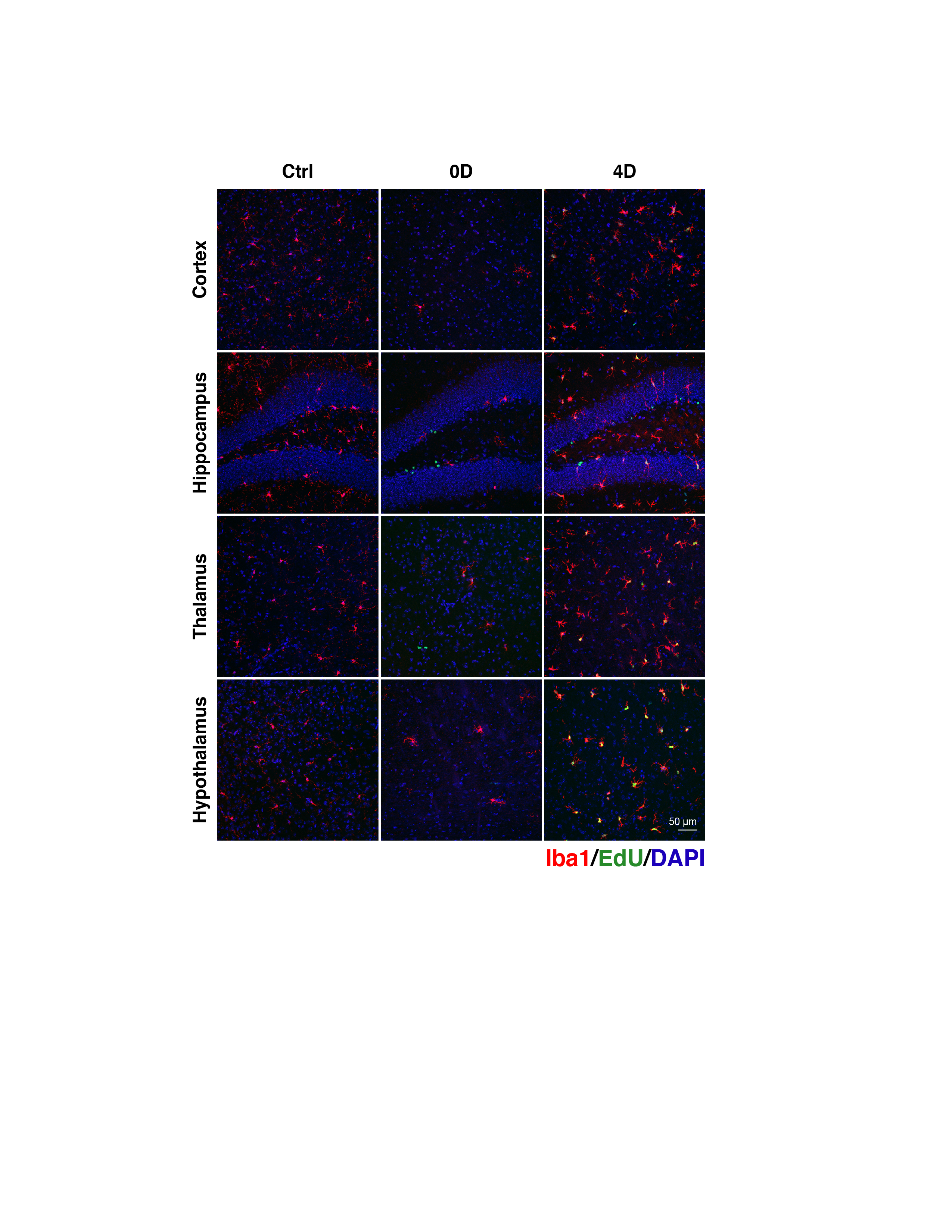

Supplement: S2 Fig — Confocal microscopy images showing microglial depletion and repopulation in different brain regions. The following markers were pseudo-colored: Iba1 (red), EdU (green), and DAPI (blue). DAPI, 4′,6-diamidino-2-phenylindole; EdU, 5-Ethynyl-2′-deoxyuridine; Iba1, ionized calcium binding adaptor molecule 1. (TIF) [file pbio.3000134.s002.tif]

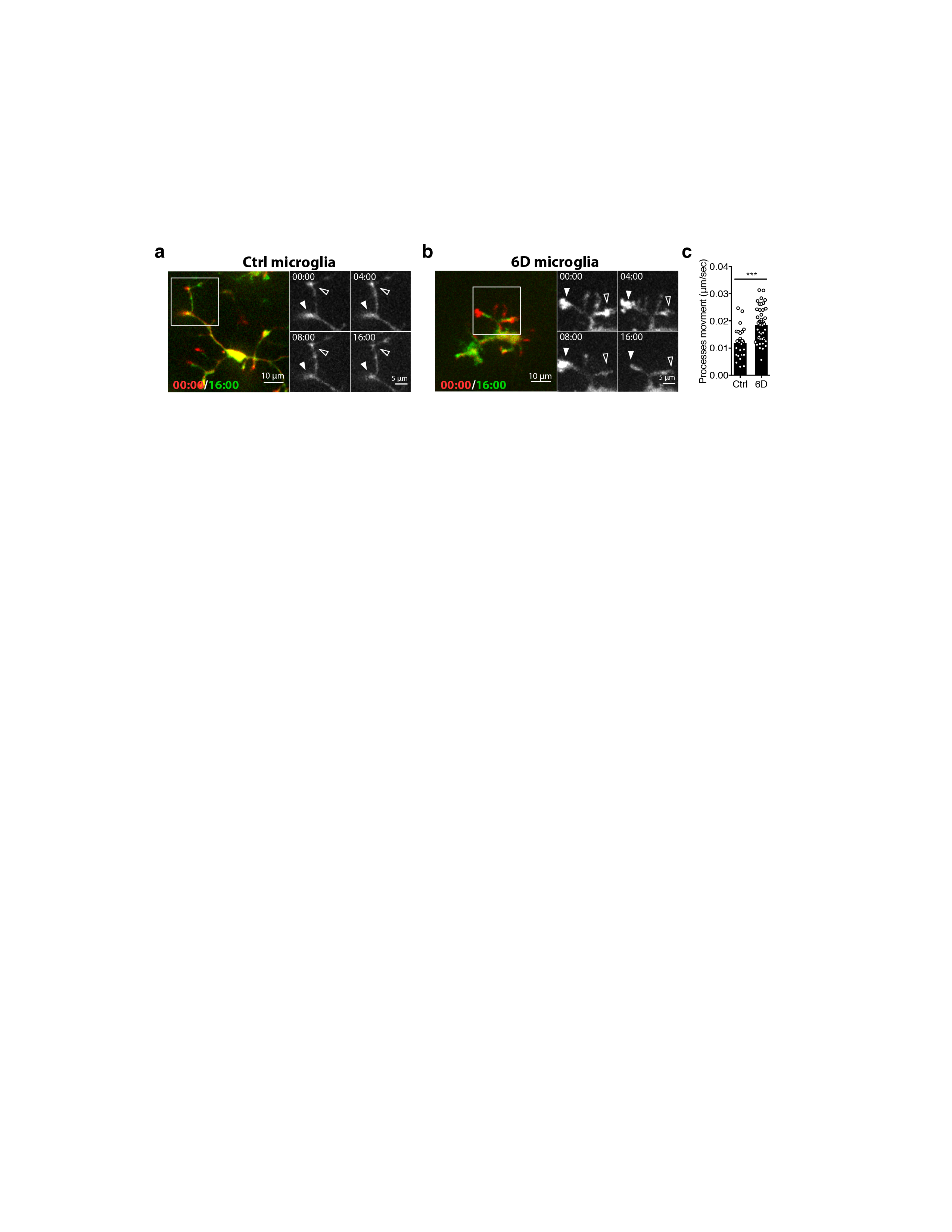

Supplement: S3 Fig — (a, b) Representative frames from live imaging of untreated control microglia (b) and microglia at day 6 of repopulation (c). Acute slices from CX3CR1eGFP/+ mice were used to image microglia. A total of 16 mins were recorded. The first frame (pseudo-colored in red) is overlaid with the last frame (pseudo-colored in green). The box highlights movement of microglial processes. Extension is indicated with closed triangles, while retraction is indicated with open triangles. (c) Quantification of the average velocity of all processes per cell in μm/sec from acute brain slices (mean ± SEM). Ctrl (n = 3 animals, 6 slices, 26 cells); 6 D (n = 2 animals, 10 slices, 42 cells). Data from each cell are plotted. Unpaired t test was applied. P value is summarized as ns (P > 0.05); *(P ≤ 0.05); **(P ≤ 0.01); ***(P ≤ 0.001); ****(P ≤ 0.0001). Individual numerical values can be found in S1 Data. CX3CR1eGFP, microglia reporter line expresses eGFP under CX3CR1 promoter; Ctrl, control; D, days. (TIF) [file pbio.3000134.s003.tif]

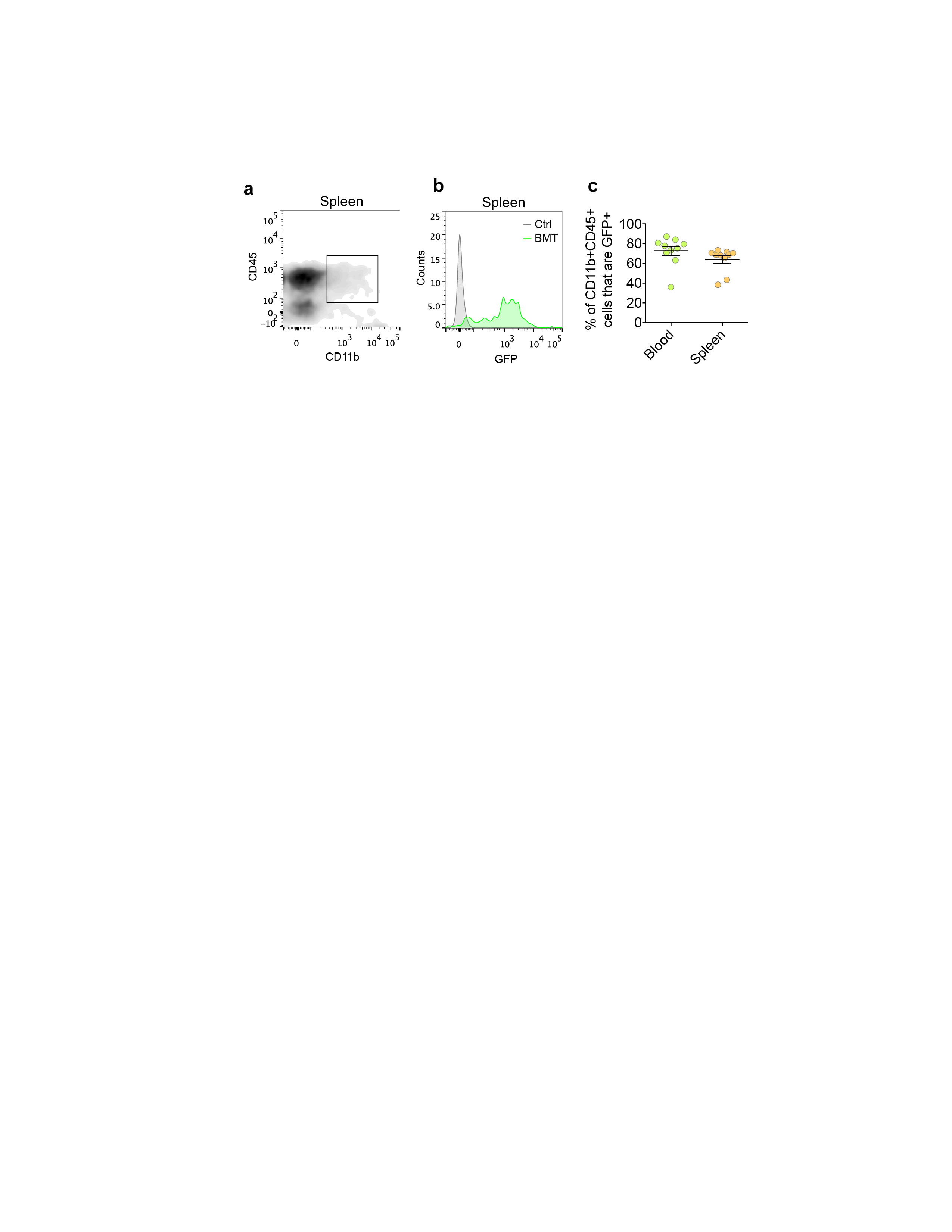

Supplement: S4 Fig — (a) Samples of the blood and spleen homogenate from the BMT mice were analyzed with FACS. Representative FACS gating plots from spleen samples are shown here. The monocytic population was selected by CD45 and CD11b and immunopositivity. Detailed gating strategy can be found in S3 Data. (b) GFP+ cells in the myeloid population were further separated and compared with the non-BMT Ctrl. (c) Quantification of bone marrow reconstitution efficiency in BMT mice. Reconstitution efficiency was defined as the percentage of GFP+CD45+CD11b+ cells out of all the CD45+CD11b+ cells. Animals used: 14 D (n = 5) and 2 Mo (n = 5). Individual numerical values can be found in S1 Data. BMT, bone marrow transplantation; CD, cluster of differentiation; Ctrl, control; D, days; FACS, fluorescence activated cell sorting; GFP, green fluorescent protein; Mo, months. (TIF) [file pbio.3000134.s004.tif]

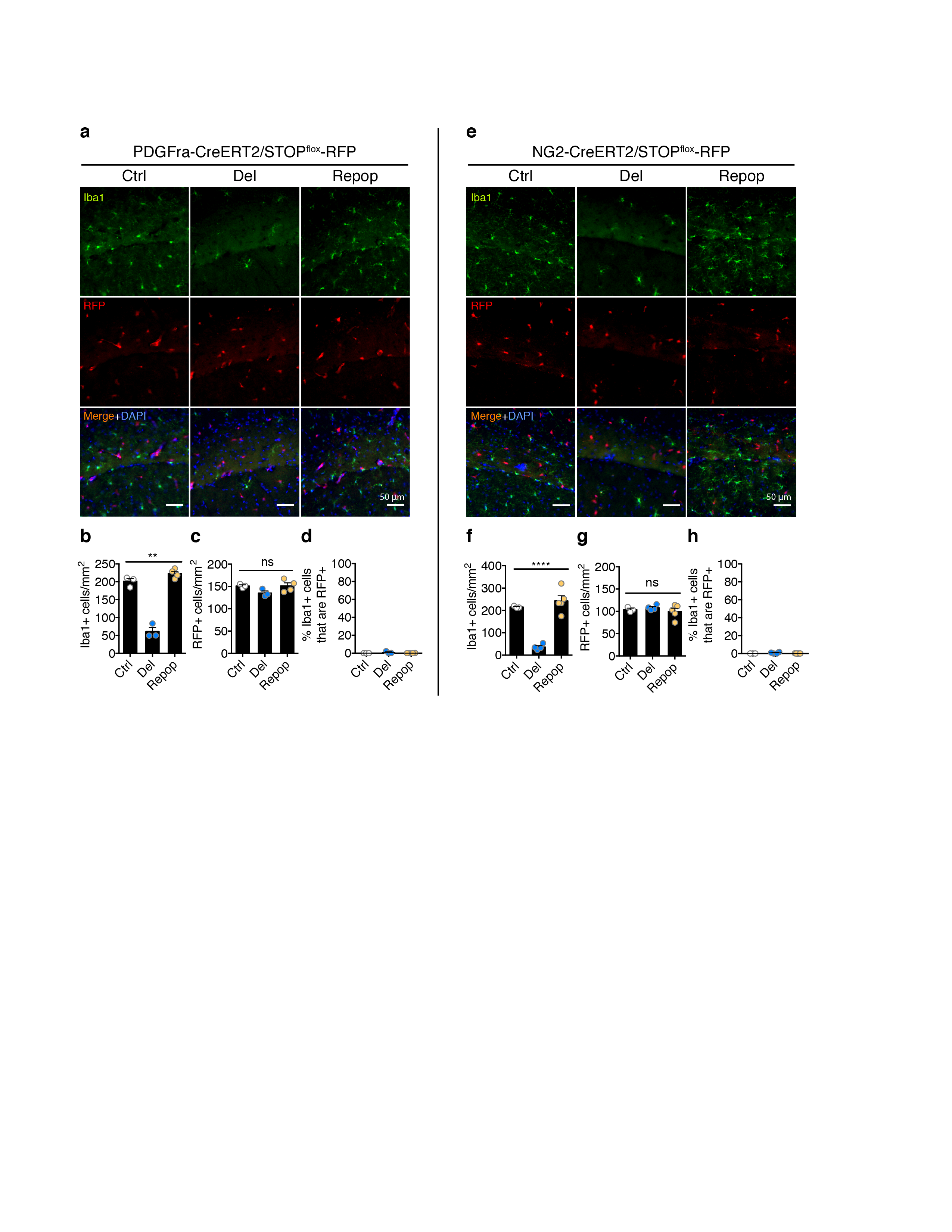

Supplement: S5 Fig — (a) Representative images of microglial depletion (PLX treatment for 2 weeks) and repopulation (normal diet for 1 week) in PDGFra-CreERT2/STOP-flox-RFP mice. Microglia are labeled with Iba1 (green). Progenitor cells from PDGFra lineage are labeled with RFP (red). (b–d) Analysis of PDGFra-CreERT2/STOP-flox-RFP mice before and after microglia repopulation. Quantification of Iba1+ microglia density (b), RFP+ cell density (c), and percentage of microglia that express RFP (d) are shown (mean ± SEM). Animals used: Ctrl (n = 3); Del (n = 3); Repop (n = 4). Kruskal–Wallis test was used for b. One-way ANOVA was used for c. (e) Representative images of microglial depletion (PLX treatment for 2 weeks) and repopulation (normal diet for 1 week) in NG2-CreERT2/STOP-flox-RFP mice. Microglia are labeled with Iba1 (green). Progenitor cells from NG2 lineage are labeled with RFP (red). (f–h) Analysis of NG2-CreERT2/STOP-flox-RFP mice before and after microglial repopulation. Quantification of Iba1+ microglia density (f), RFP+ cell density (g), and percentage of microglia that express RFP (h) are shown (mean ± SEM). Animals used: Ctrl (n = 3); Del (n = 4); Repop (n = 5). One-way ANOVA was used for statistical test. P value is summarized as ns (P > 0.05); *(P ≤ 0.05); **(P ≤ 0.01); ***(P ≤ 0.001); ****(P ≤ 0.0001). Individual numerical values can be found in S1 Data. CreERT2, tamoxifen-inducible Cre recombinase; Ctrl, control; Del, deletion; Iba1, ionized calcium binding adaptor molecule 1; NG2; neural/glial antigen 2, PDGFra, platelet derived growth factor receptor alpha; PLX, PLX5622; Repop, repopulation; RFP, red fluorescent protein. (TIF) [file pbio.3000134.s005.tif]

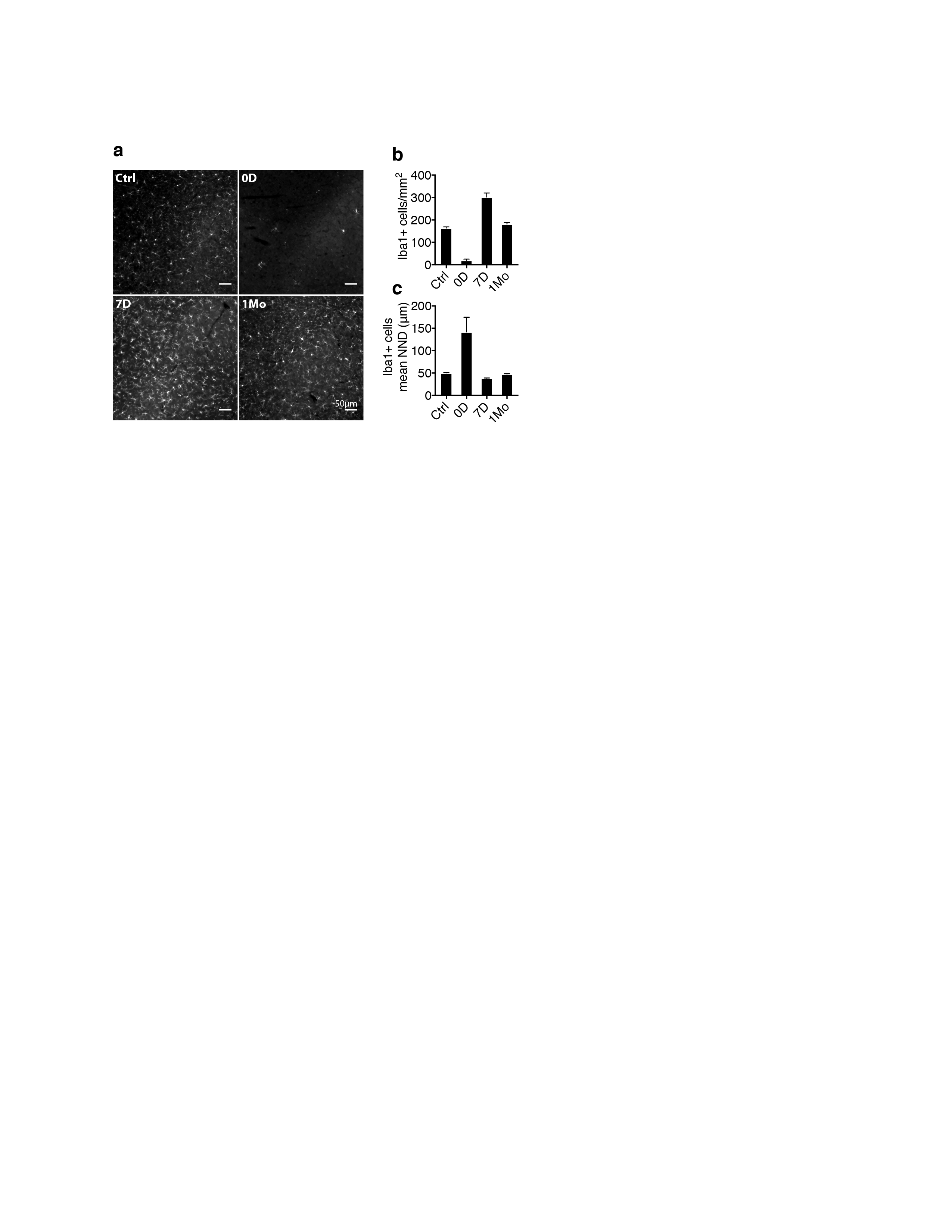

Supplement: S6 Fig — (a) Representative images showing microglial density before and after repopulation in CX3CR1-CreERT2/Brainbow mice. Mice were administered PLX5622 diet for 2 weeks before switching to normal diet and were sacrificed at various time points. Images were taken from the thalamic region. (b, c) Quantification of Iba1+ microglial density (b) and the NND of all Iba1+ microglia (c) (mean ± SEM). Animals used: Ctrl (n = 5), 0 D (n = 3), 7 D (n = 4), and 1 Mo (n = 5). Individual numerical values can be found in S1 Data. CreERT2, tamoxifen-inducible Cre recombinase; Ctrl, control; CX3CR1, CX3C chemokine receptor 1; D, days; Iba1, ionized calcium binding adaptor molecule 1; Mo, months; NND, nearest neighbor distance; PLX, PLX5622. (TIF) [file pbio.3000134.s006.tif]

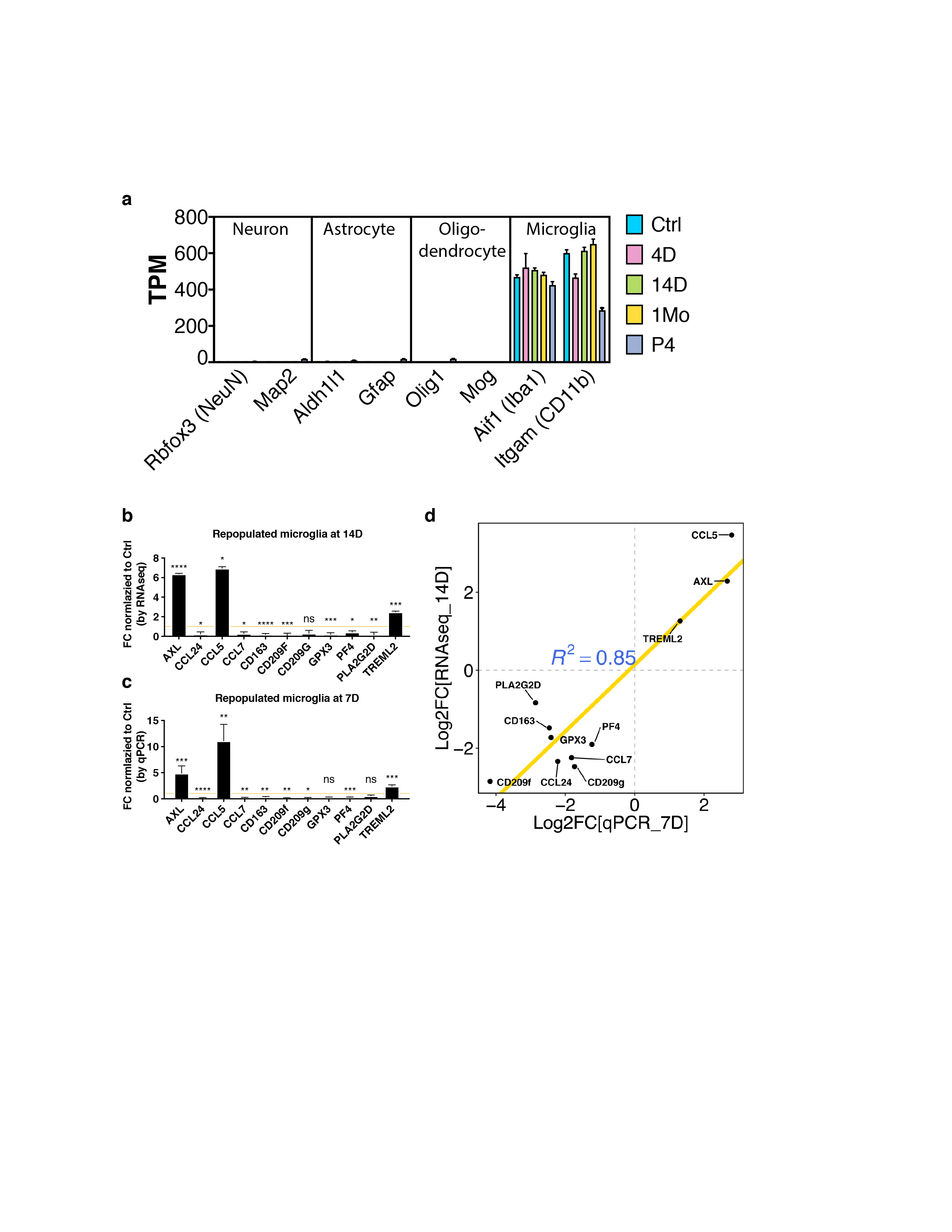

Supplement: S7 Fig — (a) Bar graph showing the presence of markers associated with major CNS cell types in the RNA-seq data. Mean value of TPM was plotted. (b) Relative expression level of selected genes from the RNA-seq data (14 D). Animals used: Ctrl (n = 4) and 14 D (n = 4). Yellow line (y = 1) represents expression level of control. (c) Relative expression level of selected genes validated by qPCR. Animal used: Ctrl (n = 11) and 7 D (n = 12). Yellow line (y = 1) represents expression level of control. Unpaired t test was used. (d) Linear regression showing the correlation between the RNA-seq data from 14 D microglia and the qPCR data from 7 D microglia (R2 = 0.8494). P value is summarized as ns (P > 0.05), *(P ≤ 0.05), **(P ≤ 0.01), ***(P ≤ 0.001), ****(P ≤ 0.0001). Individual numerical values can be found in S1 Data. CNS, central nervous system; Ctrl, control; D, days; qPCR, quantitative PCR; TPM, transcript per million. (TIF) [file pbio.3000134.s007.tif]

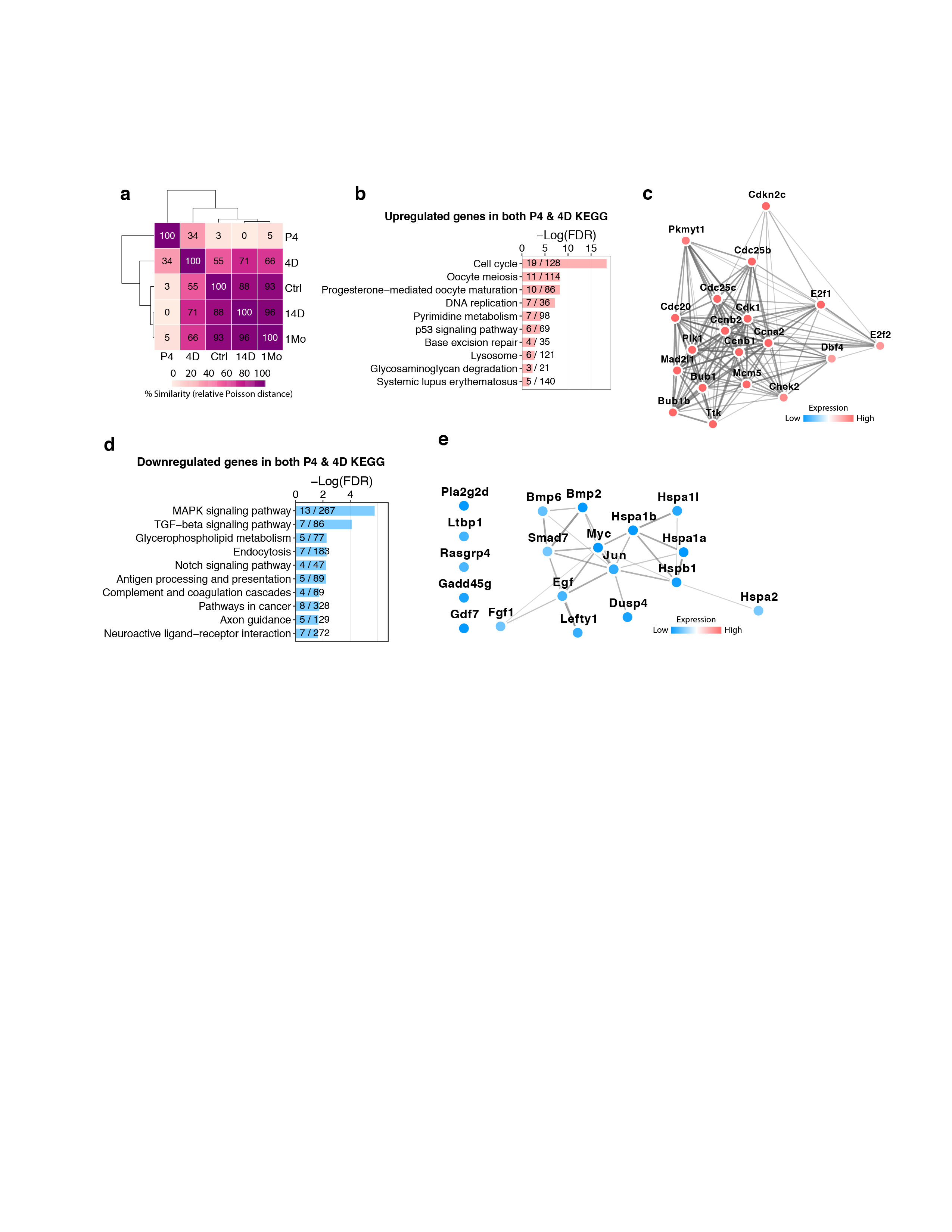

Supplement: S8 Fig — (a) Poission distance matrix showing relative similarity between each treatment group. Average Poission distance values from biological replicates were used. Relative Poission distance score is shown, which has a range of 0 (no similarity) to 100 (completely same). (b) KEGG pathway analysis using up-regulated genes found in both P4 neonatal and 4 D repopulated microglia. Top-10 enriched KEGG terms are shown. (c) Network showing cell cycle–related genes identified in pane (b). Network was constructed using STRING database. (d) KEGG pathway analysis using down-regulated genes found in both P4 and 4 D microglia. Top-10 enriched KEGG terms are shown. (e) Network showing MAPK and TGF-β signaling–related genes identified in panel (d). Network was constructed using STRING database. D, days; KEGG, Kyoto Encyclopedia of Genes and Genomes; MAPK, Mitogen Activated Protein Kinase; TGF-β, transforming growth factor β; STRING, Search Tool for the Retrieval of Interacting Genes/Proteins. (TIF) [file pbio.3000134.s008.tif]

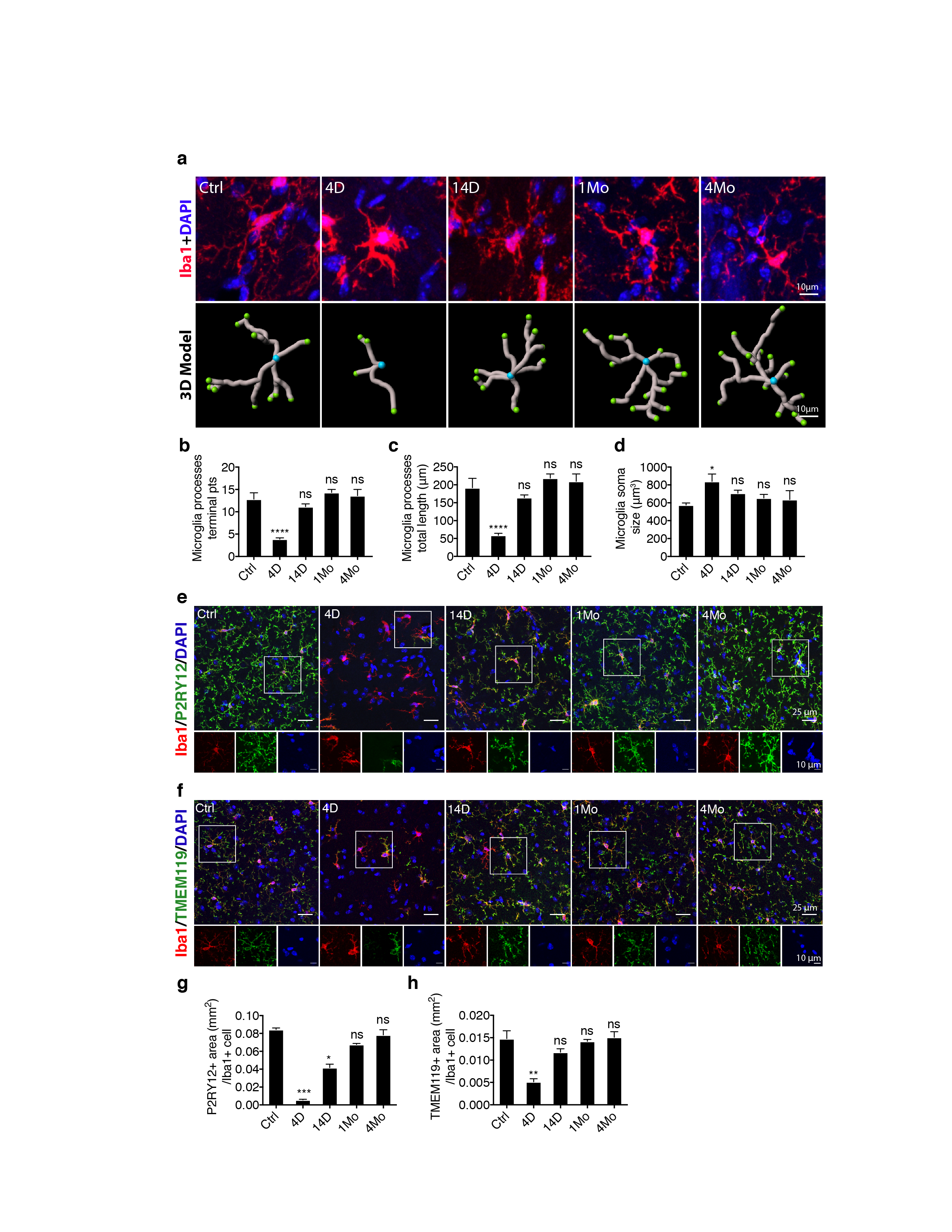

Supplement: S9 Fig — (a) Representative confocal images showing morphological features of microglia at different repopulation stages (upper) and microglia processes rendered from 3D model (lower). C57/BL6J cice (5 Mo) were treated with PLX5622 diet for 2 weeks and then switched to a normal diet at indicated time points. (b, c, d) Quantification of the number of microglia process terminal points (b), total length of the processes (c), and soma size (d). Greater than 150 microglial cells from each animal were analyzed. Mean value from each animal was plotted with SEM. Animals used: Ctrl (n = 5), 4 D (n = 5), 14 D (n = 4), 1 Mo (n = 5), and 4 Mo (n = 3). (e, f) Representative confocal images showing expression of P2RY12 (e) and TMEM119 (f) at different repopulation stages. Images were taken from the hippocampal region. (g, h) Quantification of P2RY12 (g) and TMEM119 (h) positive area per Iba1+ microglia cell number (mean ± SEM). Animals used: Ctrl (n = 5), 4 D (n = 5), 14 D (n = 4), 1 Mo (n = 5), and 4 Mo (n = 3). Statistical analyses: 1-way ANOVA with Dunnett's multiple comparisons test (against Ctrl) was used in panel (b, c); Kruskal–Wallis test with Dunnett's multiple comparisons test (against Ctrl) was used in panel (d, g, h). Individual numerical values can be found in S1 Data. Ctrl, control; D, days; Mo, months; P2RY12, Purinergic Receptor P2Y12; PLX, PLX5622; TMEM119, Transmembrane Protein 119. (TIF) [file pbio.3000134.s009.tif]

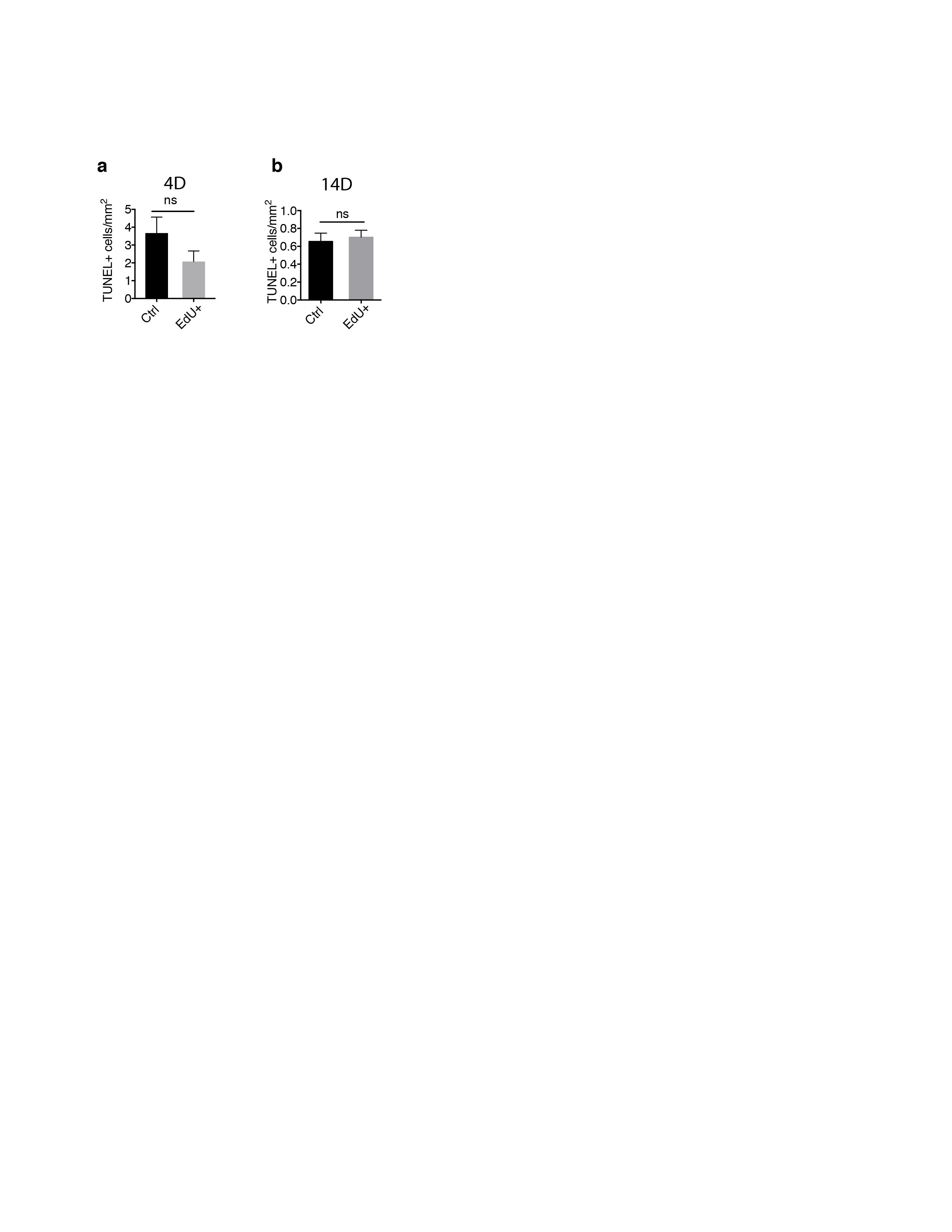

Supplement: S10 Fig — (a) Quantification of TUNEL+ cell density with or without EdU injection. EdU was injected every 24 hours via IP for 4 days of repopulation (mean ± SEM). Number of animals used for 4 D group: Ctrl (n = 5), EdU+ (n = 5). Unpaired t test was used. (b) Same analysis as in (a) with extended repopulation to 14 days. Number of animals used: Ctrl (n = 3), EdU+ (n = 4). Mann–Whitney test was used. Individual numerical values can be found in S1 Data. Ctrl, control; D, days; EdU, 5-Ethynyl-2′-deoxyuridine; IP, intraperitoneal. (TIF) [file pbio.3000134.s010.tif]

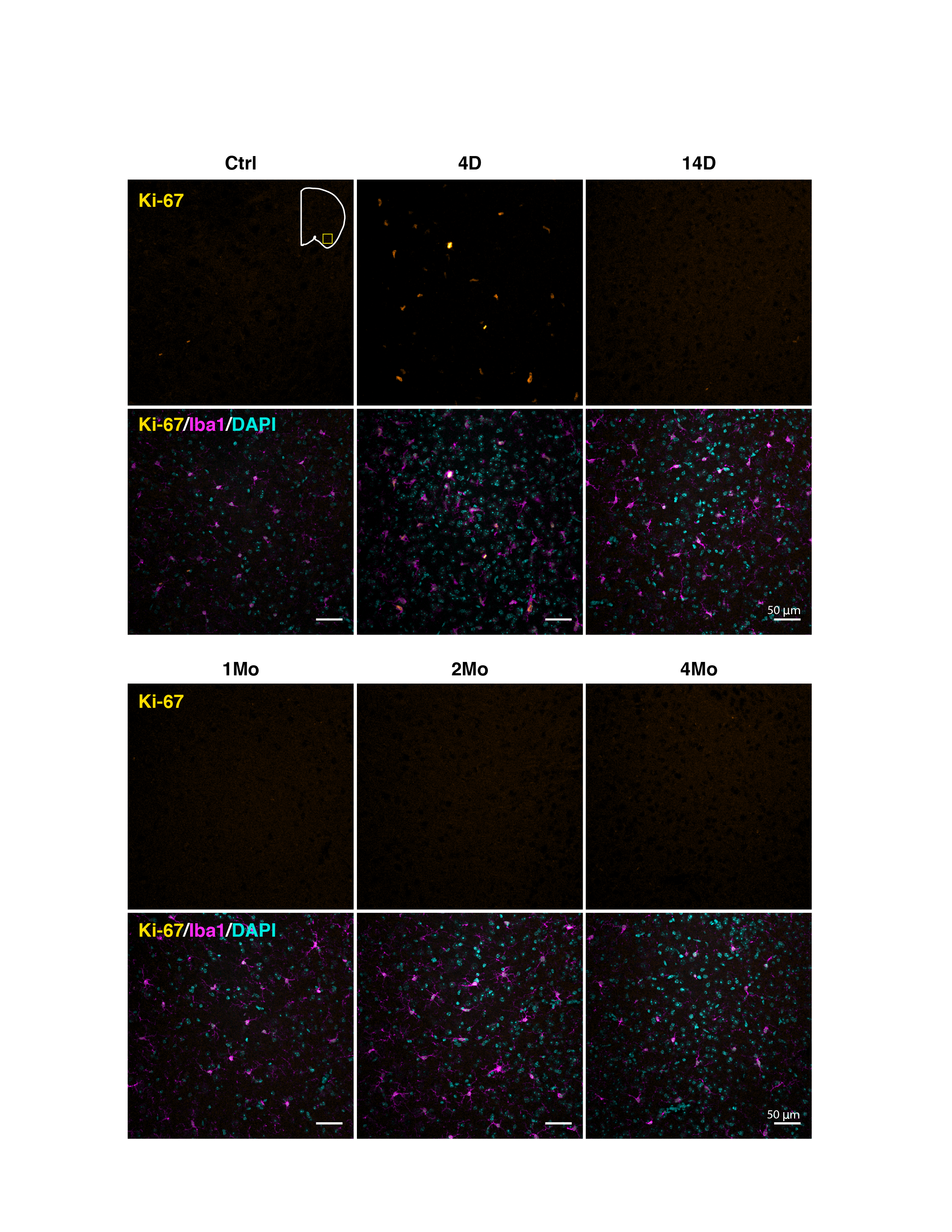

Supplement: S11 Fig — Representative confocal images showing Ki-67 (yellow) and Iba1 (magenta) staining in mice used to trace newborn microglial turnover. Images were taken from the amygdala. Only repopulation at 4 D shows noticeable Ki-67 positive staining. D, days; Iba1, ionized calcium binding adaptor molecule 1; Ki-67, Proliferation marker protein Ki-67. (TIF) [file pbio.3000134.s011.tif]

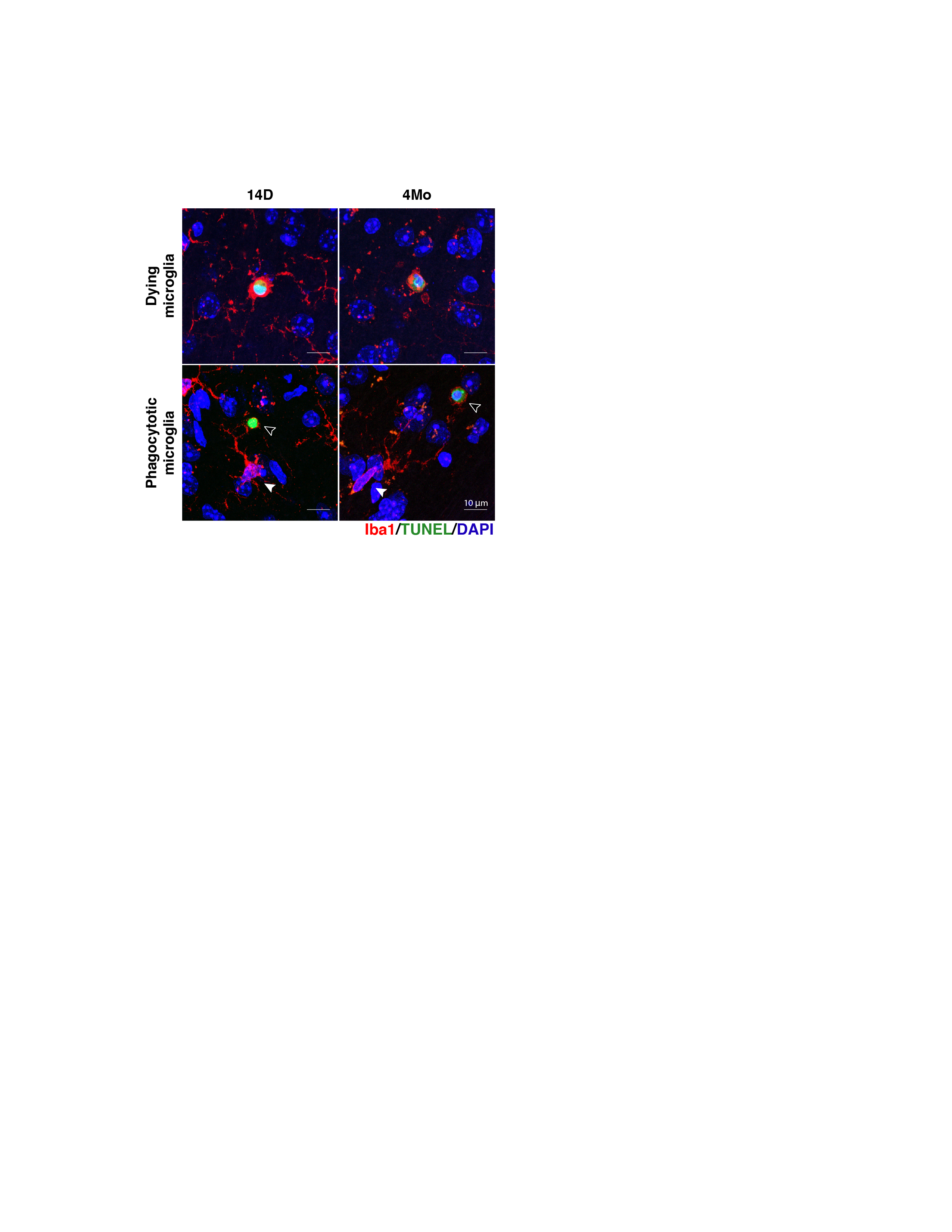

Supplement: S12 Fig — Representative confocal images showing TUNEL+ microglia and microglia forming phagocytic cup around TUNEL+ cells at either 14 D or 4 Mo. D, days; Mo, months. (TIF) [file pbio.3000134.s012.tif]

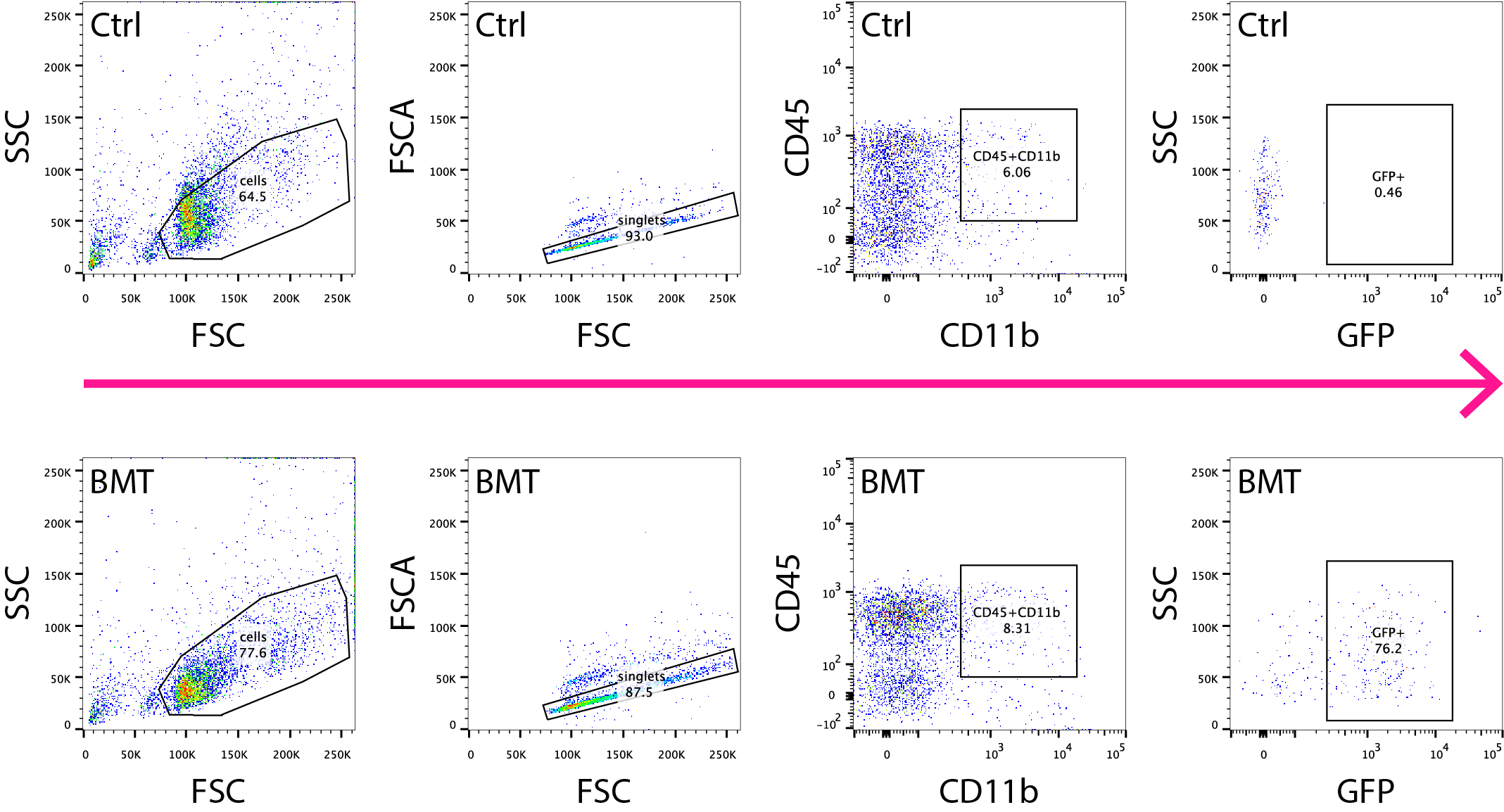

Supplement: S3 Data — BMT, bone marrow transplantation; FACS, fluorescence-activated cell sorting. (TIF) [file pbio.3000134.s023.tif]
